# Sediment source fingerprinting: benchmarking recent outputs, remaining challenges and emerging themes

**DOI:** 10.1007/s11368-020-02755-4

**Published:** 2020-09-16

**Authors:** Adrian L. Collins, Martin Blackwell, Pascal Boeckx, Charlotte-Anne Chivers, Monica Emelko, Olivier Evrard, Ian Foster, Allen Gellis, Hamid Gholami, Steve Granger, Paul Harris, Arthur J. Horowitz, J. Patrick Laceby, Nuria Martinez-Carreras, Jean Minella, Lisa Mol, Kazem Nosrati, Simon Pulley, Uldis Silins, Yuri Jacques da Silva, Micheal Stone, Tales Tiecher, Hari Ram Upadhayay, Yusheng Zhang

**Affiliations:** 1 Sustainable Agriculture Sciences, Rothamsted Research, North Wyke, Okehampton, Devon EX20 2SB UK; 2 Isotope Bioscience Laboratory-ISOFYS, Ghent University, Coupure Links 653, 9000 Ghent, Belgium; 3 Centre for Rural Policy Research, University of Exeter, Lazenby House, Pennsylvania Road, Exeter, EX4 4PJ UK; 4 Department of Civil and Environmental Engineering, University of Waterloo, Waterloo, Ontario Canada; 5 Laboratoire des Sciences du Climat et de l’Environnement (LSCE/IPSL), Unité Mixte de Recherche 8212 (CEA/CNRS/UVSQ), Université Paris-Saclay, 91191 Gif-sur-Yvette Cedex, France; 6 Environmental & Geographical Sciences, Learning Hub (Room 101), University of Northampton, University Drive, Northampton, NN1 5PH UK; 7 U.S. Geological Survey, 5522 Research Park Drive, Baltimore, MD 21228 USA; 8 Department of Natural Resources Engineering, University of Hormozgan, Bandar-Abbas, Hormozgan Iran; 9 South Atlantic Water Science Center, U.S. Geological Survey, Atlanta, GA USA; 10 Alberta Environment and Parks, 3535 Research Rd NW, Calgary, Alberta T2L 2K8 Canada; 11 Luxembourg Institute of Science and Technology (LIST), Catchment and Eco-hydrology Research Group (CAT), L-4422 Belvaux, Luxembourg; 12 Department of Soil Science, Federal University of Santa Maria, Roraima Ave. 1000, Santa Maria, RS 97105-900 Brazil; 13 Department of Geography and Environmental Management, University of the West of England, Bristol, UK; 14 Department of Physical Geography, School of Earth Sciences, Shahid Beheshti University, Tehran, 1983969411 Iran; 15 Department of Renewable Resources, University of Alberta, Edmonton, Alberta T6G 2I7 Canada; 16 Agronomy Department, Federal University of Piaui (UFPI), Planalto Horizonte, Bom Jesus, PI 64900-000 Brazil; 17 Department of Geography and Environmental Management, Faculty of Environment, University of Waterloo, EV1 Room 112, Waterloo, Canada; 18 Department of Soil Science, Federal University of Rio Grande do Sul, Bento Gonçalves Ave. 7712, Porto Alegre, RS 91540-000 Brazil

**Keywords:** Fingerprinting approach, Tracers, Biomarkers, Sediment-age dating, Weathering indices

## Abstract

**Purpose:**

This review of sediment source fingerprinting assesses the current state-of-the-art, remaining challenges and emerging themes. It combines inputs from international scientists either with track records in the approach or with expertise relevant to progressing the science.

**Methods:**

Web of Science and Google Scholar were used to review published papers spanning the period 2013–2019, inclusive, to confirm publication trends in quantities of papers by study area country and the types of tracers used. The most recent (2018–2019, inclusive) papers were also benchmarked using a methodological decision-tree published in 2017.

**Scope:**

Areas requiring further research and international consensus on methodological detail are reviewed, and these comprise spatial variability in tracers and corresponding sampling implications for end-members, temporal variability in tracers and sampling implications for end-members and target sediment, tracer conservation and knowledge-based pre-selection, the physico-chemical basis for source discrimination and dissemination of fingerprinting results to stakeholders. Emerging themes are also discussed: novel tracers, concentration-dependence for biomarkers, combining sediment fingerprinting and age-dating, applications to sediment-bound pollutants, incorporation of supportive spatial information to augment discrimination and modelling, aeolian sediment source fingerprinting, integration with process-based models and development of open-access software tools for data processing.

**Conclusions:**

The popularity of sediment source fingerprinting continues on an upward trend globally, but with this growth comes issues surrounding lack of standardisation and procedural diversity. Nonetheless, the last 2 years have also evidenced growing uptake of critical requirements for robust applications and this review is intended to signpost investigators, both old and new, towards these benchmarks and remaining research challenges for, and emerging options for different applications of, the fingerprinting approach.

## Introduction

Accelerated soil erosion and sediment delivery are widely recognized as globally pervasive threats to ecosystem services essential for sustainable livelihoods and development (Pimentel 2006; Montgomery 2007; Maetens et al. 2012; Borrelli et al. 2017; Garcia-Ruiz et al. 2017). Targeted governance and management of soil and water resources are, as a result, policy priorities worldwide (Montanarella 2015; Wu et al. 2020). Well-designed policies and control strategies for protecting soil and water resources are dependent on reliable and scale-appropriate information on the key sources of the sediment problem which is manifested in the form of both on-site and off-site consequences. With respect to scale, the river catchment continues to be seen as a fundamental landscape unit for framing both the scientific exploration of sediment systems and for devising meaningful management strategies within the context of natural hydrological hierarchies. Spatial scales for sediment sourcing studies have ranged from hillslopes to large (> 10,000 km^2^) river basins. Fine-grained sediment (< 2 mm, but frequently < 63 μm or finer in most fingerprinting studies) remains the focus of much attention, both scientifically and managerially, since finer particulates are an important vector for the transfer, dispersal and fate of nutrients and contaminants, whilst also causing detrimental impacts on all aquatic trophic levels including diatoms (Chen et al. 2019), macroinvertebrates (Gieswein et al. 2019), macrophytes (Jones et al. 2012) and fish (Kemp et al. 2011).

The most widely used direct approach for investigating catchment sediment sources is sediment fingerprinting (Collins and Walling 2004; Walling 2005; Krishnappan et al. 2009; Walling 2013b; Miller et al. 2015; Owens et al. 2016). Fundamentally, this approach is founded on the collection of target sediment and catchment source material samples and comparison of their properties or composite fingerprints to estimate the relative importance of different upstream sources. Since emerging in the 1970s through the work of Klages and Hsieh (1975), Wall and Wilding (1976) and Walling et al. (1979), both uptake and the scope of sediment source fingerprinting studies have increased dramatically (Walling et al. 2003; Haddadchi et al. 2013; Owens et al. 2016; Collins et al. 2017). Key and well-documented stages in the evolution and continued development of sediment source fingerprinting procedures over the past 40 years include the transgression from single component to composite (i.e. multiple property or tracer) signatures for source discrimination, adoption of frequentist and, more latterly, Bayesian un-mixing models for source apportionment and explicit treatment and estimation of sampling, analytical and predictive uncertainties.

More recently, however, many of the key assumptions underpinning the fingerprinting approach have been revisited, tested and challenged alongside the increased uptake for more general case study investigations. One outcome of these rapidly growing research efforts has been an expansion in both the complexity and the methodological diversity of the procedures reported in source fingerprinting studies. Such divergence in methodological detail has arisen due to the growing number of research groups working on the approach and despite the publication of some generic decision trees (Lees 1999; Foster and Lees 2000; Walling and Collins 2000; Walling et al. 2003; Collins and Walling 2004; Walling et al. 2006) throughout the period of expansion in uptake by the user community. In turn, the divergence in the methodological steps in fingerprinting operational procedures reported in the international literature raises doubts and uncertainties among new or early adopters and continues to challenge more strategic or widespread uptake of the approach as a standard tool for scientific or management goals (Mukundan et al. 2012; Collins et al. 2017). The absence of standardisation clearly reflects various drivers including scientific curiosity or opinion, available access to and budgets for field and laboratory resources and site-specific challenges due to natural and anthropogenic factors such as geology and soils or land use.

In the context of the above, this paper reviews the status quo for sediment source fingerprinting using inputs from both established investigators and independent researchers with skills or expertise considered to be highly pertinent to addressing ongoing challenges in refining and standardising operational procedures. The following sections report the continued growth of published outputs over the past 7 years, benchmark published studies since the release of a detailed methodological decision-tree at the end of 2017 (Collins et al. 2017), review outstanding issues in need of further research and scientific consensus and point to newly emerging themes in sediment fingerprinting research and applications.

## The status quo

### The continued global growth in published papers using sediment source fingerprinting

**Fig. 1:**
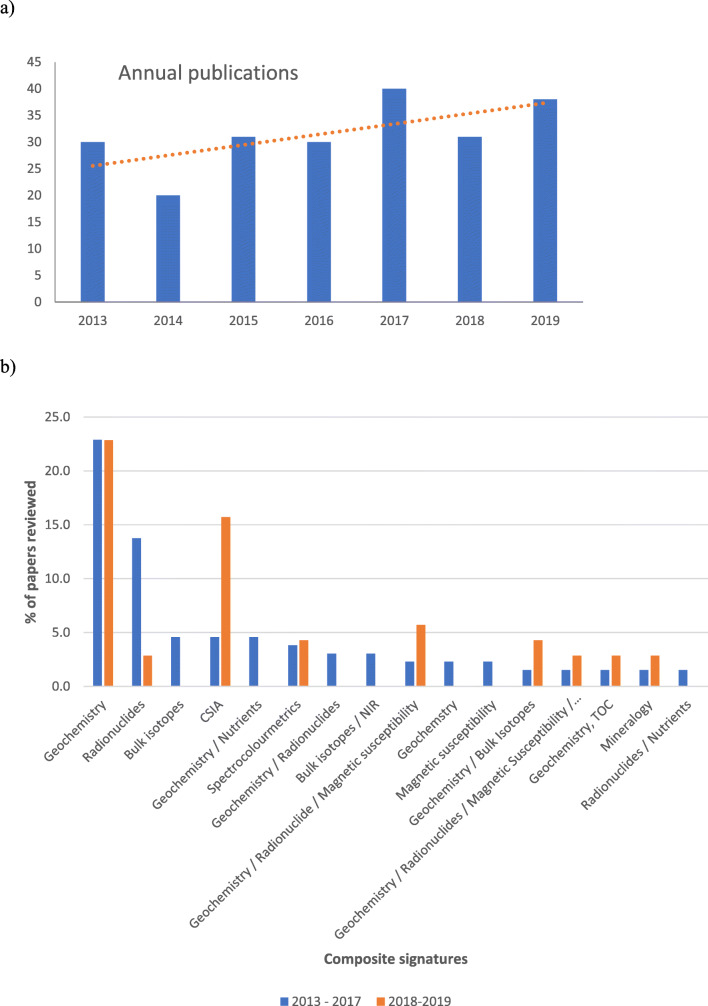
**a** Published papers reporting the use of fingerprinting each year between 2013 and 2019, inclusive (trend not statistically significant). **b** A breakdown of the composite signatures used by papers published during 2013–2017 (*n* = 131 papers) and 2018–2019 (*n* = 71 papers)

**Fig. 2:**
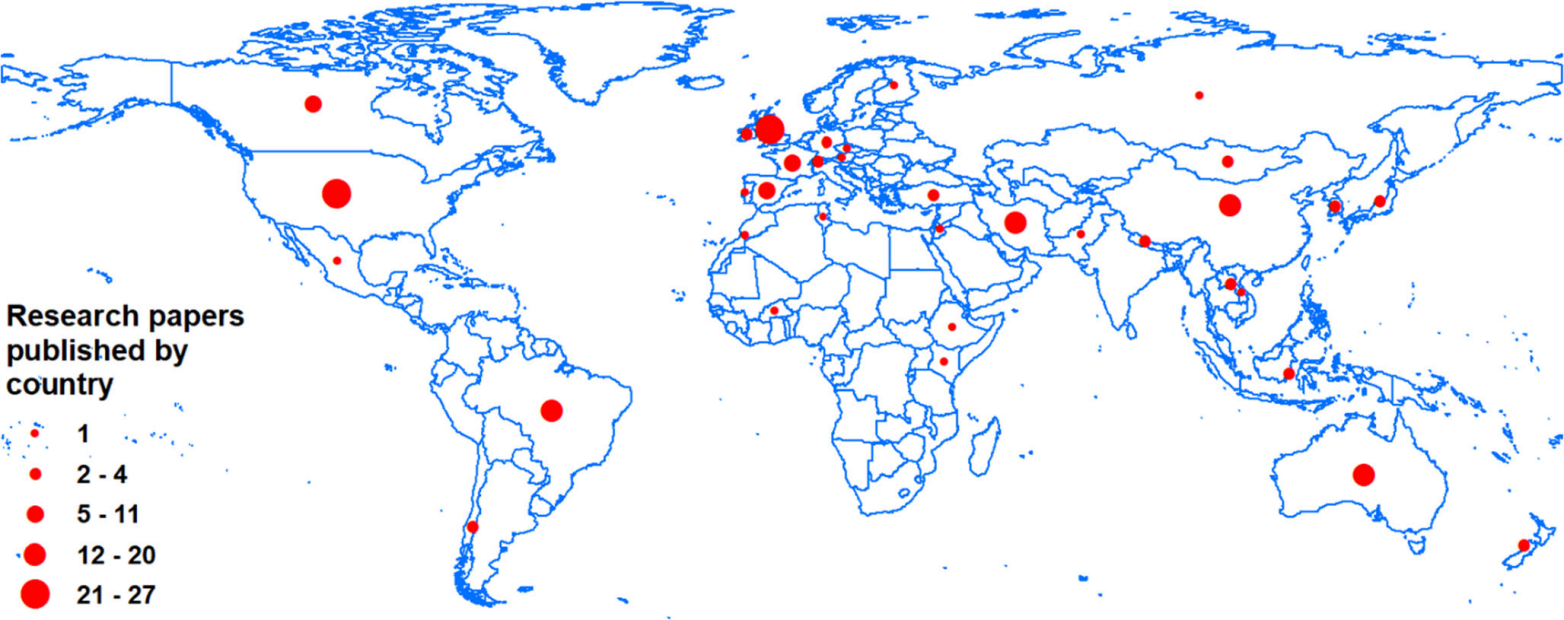
The number of fingerprinting papers published between 2013 and 2019, inclusive, using the countries for the study sites

Following on from Walling (2013b), a literature search using Web of Science and Google Scholar and the search terms ‘sediment’ and ‘source apportionment’, ‘sediment’ and ‘fingerprinting’, ‘sediment’ and ‘provenance’ and ‘sediment’, ‘source’ and ‘contribution’, suggests that the publication of papers during the period 2013–2019, inclusive, has continued to show a general upward trend, with an annual average of ~ 31 papers (Fig. 1a). Closer examination of the papers assembled by the literature search (Fig. 1b) suggests that the application of geochemical signatures remains dominant, whereas the use of fallout radionuclides has declined and applications of compound-specific stable isotopes increased. Another noteworthy trend is that the application of composite signatures combining more than one type of tracer has increased during 2018–2019. Figure 2 suggests that of the studies published between 2013 and 2019, most were undertaken in the USA, UK and China, closely followed by Australia, Iran, Brazil, Spain and France.

### Benchmarking recent papers against critical state-of-the-art methodological steps

**Fig. 3:**
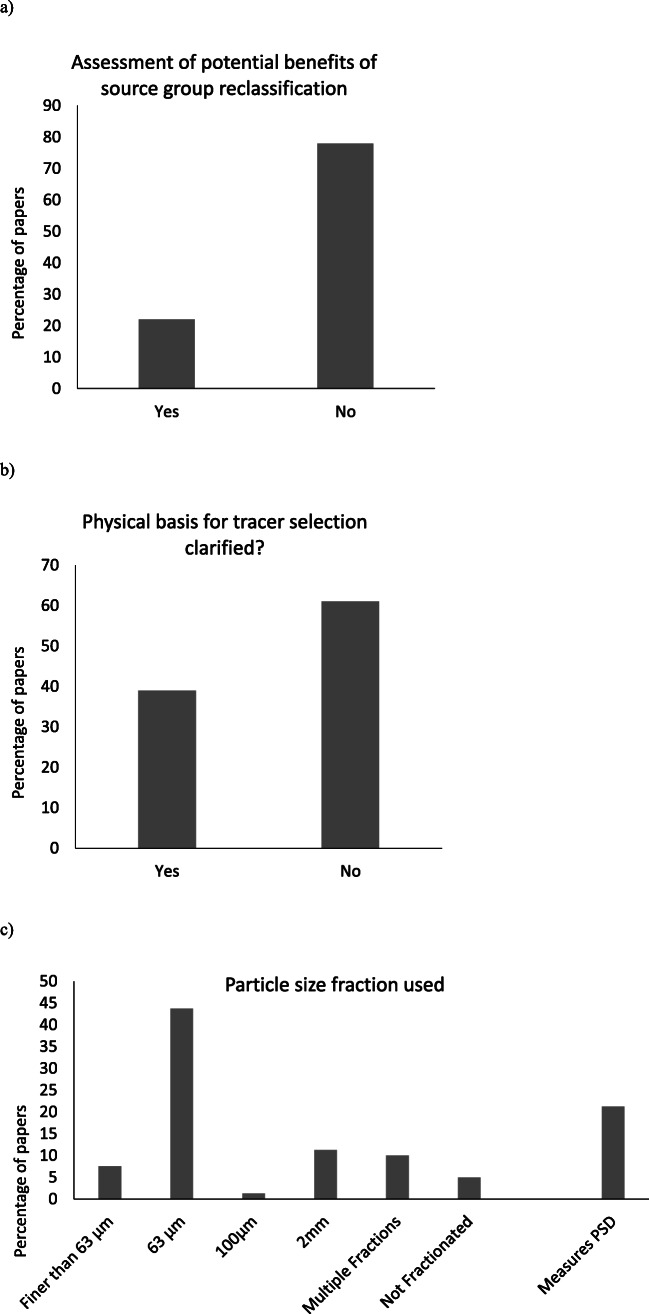

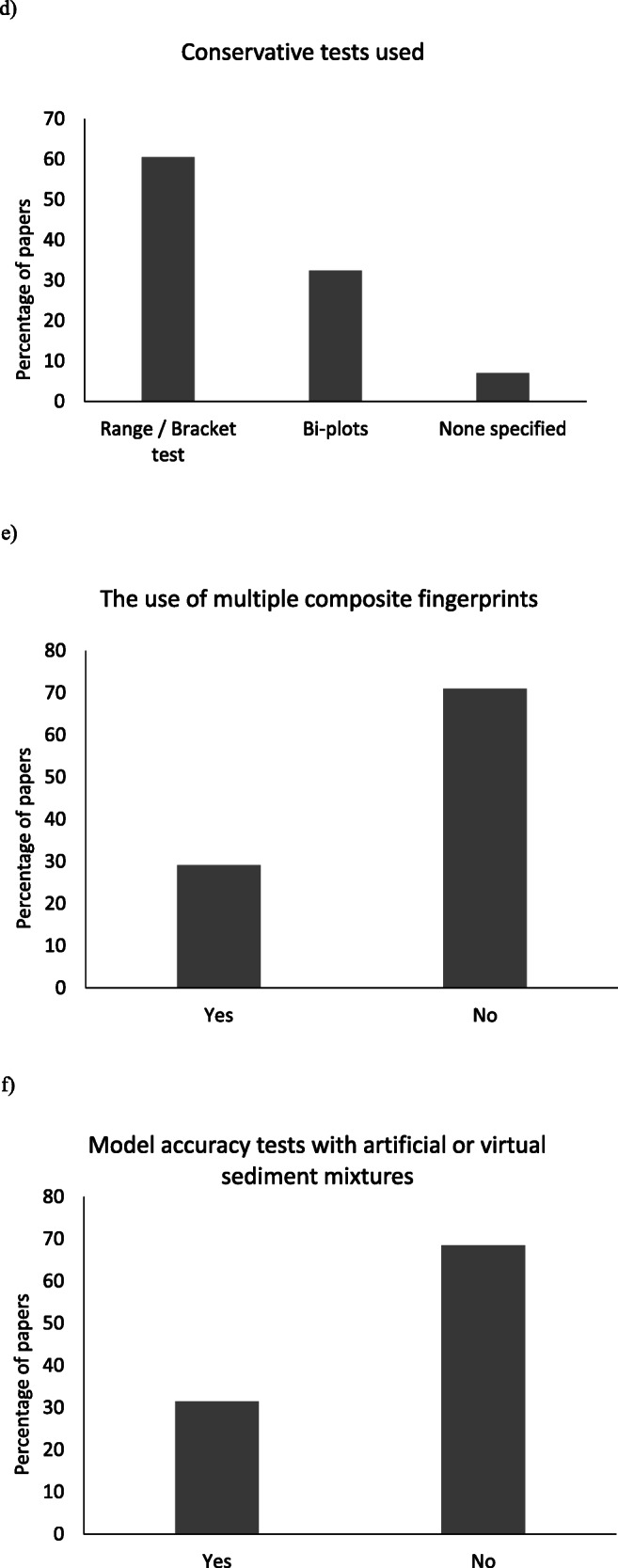
Benchmarking of recent (2018 and 2019, inclusive) papers for use of critical methodological steps

There is significant variability (Fig. 3) among the finer details of the procedures reported by international studies since the publication of a decision-tree outlining key methodological steps by Collins et al. (2017). Clearly, it takes time for recommendations to be operationalized in the international literature, but benchmarking can be used to highlight the lack of standardisation and critical gaps in some published studies. Many papers published during 2018 and 2019 do not implement essential parts of the methodology raising the possibility of major uncertainties associated with study outputs. Given the latter, a lack of standardisation among international studies risks undermining the credibility of the fingerprinting approach in the longer term. This sub-section therefore briefly revisits fundamental steps in the state-of-the-art methodology to guide authors using sediment source fingerprinting or reviewers of such work. Tang et al. (2019) recently benchmarked sediment source fingerprinting papers arising from China, and here, we extend such analysis internationally.

Sediment source classification is a critical step in the application of fingerprinting since it is used to structure the field sampling of potential sources. It is common practice to utilize a priori sources groups based upon land use. However, individual tracers might be more strongly spatially controlled by other factors such as geology. In this situation, within-source group tracer variability will likely be high resulting in more uncertain un-mixing model outputs (Pulley et al. 2015a; Pulley and Rowntree 2016). The use of multiple source group classifications can result in a greater spatial resolution of sediment provenance estimates increasing the utility of results. It should also be considered if the selected tracers can discriminate between the a priori sources or if two or more sources should be combined. In the most recent (2018–2019) publications reviewed, it remains uncommon (only 22%) for alternative source group classifications to be considered after tracers have been measured. Source groups selected a priori may be perfectly acceptable, but the exploration of multiple groupings can improve robustness.

Once source samples have been retrieved from the field, tracers for the identification of robust composite signatures need to be selected and analysed. Here, the physical basis for the discrimination of the sampled source groups is an important consideration, but only 39% of the 2018–2019 publications report some explicit assessment of this basis. Alongside the selection of the tracers, critical decisions need to be made for particle size. This has been shown to exert a strong control on source and target sediment tracer concentrations and therefore represents a major potential source of uncertainty. The decision as to which particle size range to use is commonly guided by three considerations: firstly, the primary particle size of the material being transported; secondly, the relationships between particle size and tracer concentrations (e.g. Gellis and Sanisaca 2018); and, thirdly, the time and resource requirements for sample preparation. It remains most common (59% of 2018–2019 publications) to use the < 63 μm fraction on the basis that it is generally representative of what is transported by rivers. Sample preparation using this fraction is also faster when compared with finer sizes which may require wet sieving or elutriation. However, multiple studies have shown considerable variability in tracer concentrations within this particle size range. As a result, this practice may no longer be sufficiently robust to give confidence that outputs are not heavily affected by differences in particle size between sources and target sediments. The use of narrower (e.g. < 10 μm) size ranges (10% of 2018–2019 publications) is likely to further minimize the potential for these uncertainties. It is best practice to measure the particle size distribution of the sources and target sediments and present a comparison so it can be judged if there are likely to be significant differences between the two (29% of 2018–2019 publications). Studies which fail to fractionate or use broad particle size ranges such as < 2 mm (24% of 2018–2019 publications) may not be reliable unless combined with a robust analysis of particle size effects, and therefore, caution should be exercised before their publication. It is highly unlikely that the properties of sands and silts/clays will be comparable, and sediment particle size is likely to change during sediment transport producing outputs which are primarily controlled by changes in sediment particle size rather than sediment source.

In addition to being robust discriminators, the individual properties comprising composite signatures should exhibit conservative behaviour. A conservative tracer is often determined using simple bracketing tests (Foster and Lees 2000; Wilkinson et al. 2013). Various approaches have been used to help end-users visualize this component of fingerprinting procedures (e.g. boxplot-based range test (Blake et al. 2012), point-in-polygon (Brandt et al. 2018; Bravo-Linares et al. 2018), point-in-ellipsoid (Upadhayay et al. 2018b)). Simple boxplots of source and target sediment sample tracer values provide easily understandable qualitative information on the potential transformation of the tracers being used. However, the standard range or bracket test approaches are valid for individual tracers. In contrast, the point-in-polygon approach quantitatively enables end-users to evaluate whether target sediment samples are within or outside the end-member polygon for up to three-tracer systems (Smith et al. 2013b). More recently, Upadhayay et al. (2018b) applied the point-in-ellipsoid approach that can deal with multiple tracers simultaneously. This involves the transformation of the ellipsoid for the source and target sediment tracers into circles and overlapping these circles to provide qualitative information as to whether each sediment sample is in or out of the mixing ellipsoid (Jackson 2016).

The assessment of tracer non-conservatism (92% of 2018–2019 publications) is conducted conventionally using a range test. The basic test, as noted above, determines if the tracer concentrations of the sediments fall within the maximum and minimum of the sources (Foster and Lees 2000). A stricter version of this test uses the mean tracer concentrations of the source groups and target sediments (Wilkinson et al. 2013). This test whilst being an essential part of the methodology (73% of 2018–2019 publications) is, however, unable to detect small changes in tracer concentrations which can have significant effects on source apportionment estimates. Accordingly, bi-plots have been used as a more sensitive conservatism test (Oldfield and Wu 2000) and are becoming increasingly used by recent international studies (39% of 2018–2019 publications). Bi-plots are particularly sensitive to tracer non-conservatism when two tracers are correlated in the source groups, and it can be determined if the relationship between the two is maintained in the target sediments. Here, there is considerable overlap between particle size selection and tracer conservatism as if the particle size distribution of sources and sediments is significantly different; it would be expected that a significant number of tracers would fail a conservatism test. Whilst the fractionation of source samples into a range of particle size fractions can be combined with bi-plots to attempt to separate these effects, such methods are highly time and resource intensive (Pulley et al. 2015b). Although a single composite fingerprint which achieves strong discrimination can be adequate to estimate robust results, the use of multiple different composite fingerprints can give greater assurance that tracer non-conservatism or poor discrimination is not causing uncertainties in fingerprinting results and this approach is recommended. In the 2018–2019 studies reviewed here, however, the use of more than one composite fingerprint remains uncommon (27% of publications).

Following the above critical steps for robust source discrimination, an un-mixing model is used for source apportionment. Here, it is of particular concern that 5% of the 2018–2019 publications reviewed lacked even a Monte Carlo uncertainty routine which has been increasingly seen as standard since introduced by Franks and Rowan (2000). The use of artificial (Haddadchi et al. 2014; Upadhayay et al. 2018a; Gaspar et al. 2019; Uber et al. 2019) or virtual (Palazón et al. 2015; Pulley and Collins 2018) sample mixtures for evaluating un-mixing model predictions is strongly recommended, regardless of tracer types. However, their use remains limited (29% of 2018–2019 publications). Physical mixtures of source samples have some potential advantages, compared with virtual mixtures, since the former are designed to mimic the mixing of source-specific sediment. Real artificial mixtures do, nevertheless, require greater resources in terms of staff time and laboratory budgets. The mixing and splitting of source samples for combination into artificial mixtures can introduce errors. Virtual mixtures may be as simple as putting source group medians through the un-mixing model to represent a 100% contribution from each source or taking the mean of two sources to represent a 50% contribution from each. Where differences between the texture distributions of the source samples used to generate the source-specific tracer concentrations being mixed into virtual target sediment mixtures are pronounced, the use of such mixtures can encounter problems associated with bias. Equifinality can render an un-mixing model mathematically unsound where there are multiple combinations of different source contributions which can produce the tracer concentrations measured in a target sediment sample. Mixtures are a key method for the assessment of this source of uncertainty. It is commonly considered that n-1 tracers for the number of source groups are required in a composite fingerprint. However, in practice, it is often not fully appreciated that each of the sources must be discriminated strongly by at least one tracer. This is especially of concern when multiple source groups (> 3) are used as it is unlikely that sufficiently strong discriminators are available for all sources. Here, it is recommended that mixtures of a 100% contribution from each source are trialled as an absolute minimum to ensure that each source is recognized by the un-mixing model before a study is considered suitably robust. Goodness-of-fit (GOF) has long been used to assess the reliability of modelling outputs. However, recent work (Gaspar et al. 2019) using synthetic sample mixtures has shown that GOF has little relation with model accuracy and therefore a high GOF cannot be accepted alone as robust evidence that model predictions are accurate. Instead, synthetic (actual or virtual) mixtures represent a more state-of-the-art and robust method of evaluating un-mixing model results.

A critical decision for fingerprinting studies concerns the sampling of target sediment, i.e. the sediment that is being apportioned and, which should be representative of the study catchment. Target sediment can include suspended, channel bed, floodplain and reservoir or lake sediment. Critically, the choice of the type of target sediment depends on the specific objectives of the study and, for this reason, this element has not been benchmarked. If understanding how sediment sources change through storm events or between events is of interest, then storm samples of suspended sediment should be collected (Mukundan et al. 2012; Gellis et al. 2015). Suspended sediment can be collected using several approaches: manual samples, automatic pump samplers and passive samplers (Phillips et al. 2000). Bed sediment reflects sediment that is eroded and deposited over several events and has been used to source sediment over time periods of weeks to months or years (Miller and Orbock-Miller 2007). Floodplain sediment, which has been used in sediment fingerprinting, is deposited during larger flow events that may occur at a frequency of years (Miller et al. 2015). If the objective is to obtain a target sample that is representative of long-term conditions in the order of years, lake or reservoir sediment may be more appropriate. Lake cores have been used for understanding sediment sources over historic and geologic time scales (Foster et al. 1998).

## Outstanding issues requiring further research and consensus

The following sections review a number of essential topics related to sediment source fingerprinting that require further work and consensus. These topics emerged during the initial discussions between the authorship team over the detail of this new paper and reflect critical research or standardisation gaps.

### Spatial variability in tracers and sampling implications for end-members

The spatial variability of tracers across scales (e.g. at an individual end-member sampling location or at larger scales across each end-member sampled within the catchment in question) and the scope to capture this with different sampling strategies commonly employed in sediment fingerprinting studies merit further investigation. To date, only a few studies have explored tracer spatial variability at an individual sampling location representative of a particular source end-member (e.g. Du and Walling 2017; Pulley and Collins 2018; Collins et al. 2019). The same is true for spatial variability over larger scales (Wilkinson et al. 2015). Accordingly, there remains a need to expand existing work to include more tracers and to confirm the sensitivity of source apportionment estimates to different sampling strategies. There is a rich history of use of spatial statistics in soil science (McBratney et al. 2003; Lark 2012; Minasny and McBratney 2016), where models for spatial exploration, prediction, classification, sample design/re-design and improved regression inference have all been constructed, and clearly, the sediment fingerprinting community should interact more with such expertise. In the case of the former, a field will often represent an individual sampling location for a given (e.g. land use category) end-member. Here, in an ideal world, the commonplace practice of bulking replicate samples from the same field into a single composite should be avoided, as it provides no assessment of microscale tracer variability, but resources do not support this idealism. Critically, it is not guaranteed that a bulked value is equivalent to the mean of separately analysed replicate samples, wherein a measure of variance is possible. Spatial configuration of the sampling is also important with a ‘W’ (sampling along a W-shaped zig-zag path), transect or random sampling protocol, all being commonplace. The orientation of the W or transect should try to traverse all major sources of within-field variation, whilst also recognising any obvious connectivity pathways. If preferential (e.g. clearly visible erosion hotspots) sampling is undertaken, it should be consistent across all fields/locations of the target landscape and across all tracers, so resultant biases are interpretable (e.g. consistent over-prediction or under-prediction, but not mixtures of both), especially when it comes to implementing the un-mixing models for source apportionment. Where resources permit, a pilot sampling strategy can be used to inform subsequent sample re-designs to capture maximum tracer variability at a minimum cost. Re-designs can be further refined to cater optimally for any desired preferential sampling of areas (e.g. highly connected slope-to-channel pathways), times and depths of most interest for a given tracer or group thereof. Over a period of time, and provided far more studies followed similar two-stage sampling protocols (pilot-to-optimally re-designed), it is likely that useful ‘rules of thumb’ would evolve for a range of spatial tracer designs and thus negate the need for many pilot studies. Here, the concept of ‘external objectivity’ (Matheron 2012) is noteworthy, where the value of a given statistically robust methodology can be assessed by its performance in the ‘long run’ through an increasing number and variety of applications.

### Temporal variability in tracers and sampling implications for end-members and target sediment

Catchment sources can be dynamic environments, and, consequently, the potential for their fingerprints to vary temporally should be explored. Even if exposed to the same climatic conditions, end-member fingerprints are subject to biotic and abiotic factors that result in temporal changes (e.g. Lauber et al. 2013; Collins et al. 2019). Still, we have a limited understanding of the magnitude of those changes and their predictability and of how the temporal variability of end-member fingerprints compares with the spatial variability within and between end-members. Independently of the temporal scale of investigation, from individual storm runoff events to decadal timeframes (see Laceby et al. 2019), end-member sampling is often limited to a single sampling campaign. The main reason for this being that robust evaluation of temporal variability requires repeat sampling and thereby has resource implications which are often prohibitive in the context of research budgets.

Collins et al. (2019) investigated if δ^13^C, δ^15^N, TC and TN could be used to discriminate top and sub-soil at field scale. The authors combined assessment of potential spatial and temporal variations in soil properties and found that all tracers exhibited some statistically significant temporal variation. Overall, the results suggested that temporal variation might also be relevant in sediment fingerprinting studies conducted at larger scales. Reiffarth et al. (2019) investigated the potential of compound-specific stable isotopes (CSSIs) to trace soils derived from different cultivated fields. The authors reported that variability in δ^13^C FA values increased in fall and spring, which could affect the number of sub-samples required per source. They argue that more research is required to investigate intra- and inter-annual isotope tracer variation (i.e. tillage effects and seasonality). Special attention should also be made in study sites where large events are likely to occur during the period of investigation (e.g. typhoons; see Chartin et al. (2017) and Evrard et al. 2019a) or where anthropogenic disturbance might cause significant impacts over a short time period and result in more pronounced temporal variability in end-member signatures.

Sediment origin can exhibit significant variations both within and between storm runoff events (Carter et al. 2003; Nosrati et al. 2018), and high temporal resolution sediment tracing can provide useful information not only on phasing between flow and sediments during events but also on potential sources that affect different stages of the hydrograph (e.g. Vale and Dymond 2020). However, despite the need to obtain reliable and high-frequency information on target sediment fingerprints, studies are often hampered by difficulties in obtaining samples of sufficient quality and quantity for laboratory analyses and by analytical limitations and costs (Horowitz 2013; Conn et al. 2016). This, in turn, restricts high-frequency sampling campaigns to a limited number of events. During the last decade, progress in environmental monitoring has facilitated the collection of hydrochemical data at high frequency (e.g. minutes), including nutrient concentrations (i.e. C, N, P), species (e.g. NO_3_, NO_2_, NH_4_) and composition (e.g. DOM); see Blaen et al. (2016) and Ruhala and Zarnetske (2017) for reviews. These newly gained datasets have significantly improved our mechanistic understanding of catchments (Rode et al. 2016), but we are still limited by the significant uncertainties in hydrological observations (Beven et al. 2020). In sediment source tracing, a next leap forward in environmental monitoring is to develop field deployable, robust and affordable sensors for measuring sediment fingerprints at high temporal resolution and over long periods of time (Horowitz et al. 2015). There are many advantages of using field deployable instruments to estimate suspended sediment composition in situ, including (i) shorter periods between monitoring and generation of results, (ii) elimination (or reduction) of sample preservation and transport issues experienced with conventional auto-sampling approaches, (iii) the possibility of acquiring fast and reliable data about pollutant levels in emergency situations, (iv) reduction of energy consumption (when compared with laboratory analyses) and (v) reduction of reagent use and waste (Galuszka et al. 2015 and references therein).

Recently, Martinez-Carreras et al. (2016) demonstrated that absorbance data measured with a spectrophotometer can be used to estimate suspended sediment properties reliably. The authors installed a spectrophotometer at the outlet of the Weierbach catchment (Luxembourg; 0.45 km^2^) and measured stream water light absorbance. They then measured percentage weight loss-on-ignition (LOI) on suspended sediment samples collected fortnightly during the monitoring period and during a storm runoff event to calibrate a regression model able to predict suspended sediment LOI from light absorbance. Lopez-Roldan et al. (2016) successfully predicted the contribution of the water origin to the Barcelona drinking water network using data from a spectrophotometric probe and a small number of physico-chemical parameters, whereas Noij and Bobeldijk (2003) used data from a spectrophotometric probe to detect the intrusion of chemical and microbiological constituents in a water network. Current investigations are addressing the potential use of high-frequency absorbance data for sediment source tracing (Lake et al. 2019), but many challenges are still to be addressed before absorbance can be routinely used for tracing, including assessment of tracer conservatism and influence of particle size distributions. Such sensors limit available tracers and thereby may not be suitable for fingerprinting sources in some catchments. Accordingly, the use of UV-VIS sensors should be seen as complementing, not replacing, traditional grab sampling campaigns (Sobczak and Raymond 2015). It will always be necessary to collect some samples periodically to calibrate a sensor, to provide cross-checks to detect unreliable instrument readings and to validate the raw spectra before use (Gamerith et al. 2011). Moreover, system stationarity should not be assumed and calibrations should be controlled regularly (Horowitz 2013).

### Tracer conservatism and knowledge-based pre-selection

One of the key requirements in a successful sediment fingerprinting exercise concerns the selection of a combination of tracers for source apportionment (e.g.Walling et al. 1993; Gellis and Walling 2011; Walling and Foster 2016; Collins et al. 2017). Within this context, a tracer can be the concentration of an inorganic (e.g. Al, Ti, Li, radioactive or stable isotopes, elemental ratios, mineralogy), or an organic (e.g. *n*-alkanes, fatty acids, pharmaceuticals, disinfection by-products) constituent or a physical measurement (e.g. particle size, density, magnetic susceptibility, colour). A useful tracer, regardless of type, has to meet two specific criteria: (1) it must be able to uniquely identify and differentiate between potential sources, and (2) it must be conservative (stable) during the transit time from its source point to where it was collected as part of a target (e.g. suspended, bed, lake core) sediment sample, downstream of all the potential sources in a catchment (e.g. Foster and Lees 2000; Collins et al. 2017).

In the context of sediment source fingerprinting, the term conservative means that the concentration/measurement will remain unchanged, at least within sampling/analytical/measurement error, as the mobilized material traverses and is routed through the system under evaluation. Tracer properties may change at different stages of sediment transfer trajectories from source to sink. Here, there is the potential for two possible scenarios: (1) tracers may transform before the mixing of material mobilized and delivered from the individual sources and/or (2) after mixing of the source-specific mobilized material into the target sediment. Whilst the potential sources of sediment within different catchments tend to be fairly similar, their relative contributions to the target sediment sampled tend to be system-specific. As such, the selection of an appropriate set of tracers for a particular system can represent an extensive and resource intensive watershed-specific trial-and-error exercise.

Although both criteria (differentiation and conservatism) are equally important for tracer selection, they are presented in the order in which they typically would be applied during a fingerprinting exercise. Further, more tracers are likely to meet the first criteria (differentiation) than to meet the second one (conservatism). Hence, conservatism (stability) is likely to be more limiting in tracer selection than discrimination between potential sources (e.g. Foster and Lees 2000; Collins et al. 2017). Conservatism results from the level of chemical/geochemical/mineralogical stability associated with each potential tracer or group of tracers. In various aquatic environments, that stability is a function of a tracer’s chemical reactivity in response to the range of physico-chemical and biological conditions extant. On that basis, among the categories of potential tracers, stability is likely to rank, from higher to lower as follows: physical measurements > inorganic constituents > organic constituents. Hence, all things being equal, the utility of potential tracers probably should be evaluated in that order. This general rule-of-thumb should apply in catchments characterized by typical potential sources such as geological units, managed forests, cultivated hillslopes, intensively managed pastures, channel banks, road cuttings/surfaces/verges and instream (secondary) sources. Alternatively, in catchments containing atypical sources such as specific extraction operations (e.g. coal, gold, base metal mining), urban areas or a particular manufacturing/processing facility (e.g. pharmaceuticals, galvanizing plant, smelter, petrochemicals, sewage treatment), appropriate tracers may be self-evident, regardless if the tracer is a physical measurement or a chemical constituent (e.g. Horowitz and Stephens 2008).

Assessment of tracer conservatism is both a critical element of state-of-the-art procedures and an outstanding issue, since it is accepted and widely reported that current standard tests remain a black-box approach (Koiter et al. 2013a). Limited work (e.g. Motha et al. 2002) to date has investigated tracer conservatism explicitly, and case studies continue to apply conventional tests. Regardless of the test used for tracer conservatism, when the target sediment samples do not pass these tests, there are two possibilities: (1) missing sources and/or (2) enrichment/depletion of tracer values due to various processes including, for example, hydrodynamic sorting or biomarker degradation/addition. In other words, that particular tracer or set of tracers is behaving non-conservatively. The international sediment fingerprinting community is aware of the impact of non-conservative tracers on the uncertainties associated with estimated source contributions (Cooper and Krueger 2017); however, there is no formal agreed test to detect tracer transformation during sediment generation, delivery, post-deposition and collection. On this basis, expert opinion is inevitably crucial and one of the key aspects of selecting the most suitable and reliable tracers for sediment source apportionment.

#### Inorganic tracers

End-users of sediment fingerprinting should be aware that different analytical procedures are likely to produce different concentrations for specific sediment-associated constituents (tracers); this is particularly true for matrix-bound inorganic elements because total (≥ 95% recovery of what is present) quantitation typically depends on completely solubilizing the sediment particles prior to analysis (e.g. Horowitz 1991, 2013). Whilst this analytical approach ensures complete quantitation of matrix bound constituents, that should not be taken to mean that this value actually will be the most useful in differentiating between potential sources within a catchment. In some cases, specific partial extractions prior to chemical analysis (e.g. Chao 1984; Horowitz 1991; Hall and Pelchat 1999) might produce more useful values for differentiating sources. Final decisions, like tracer selection, regarding methods of quantitation may turn into a trial-and-error exercise for each study area.

**Fig. 4:**
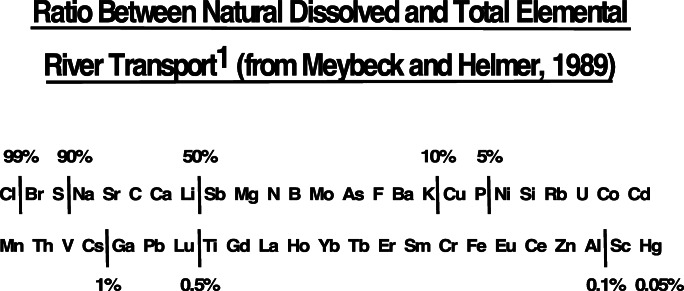
The relative solubility (hydrophobicity) of selected inorganic constituents in natural waters. The lower the solubility, the greater the stability. Stability increases from the lower right to the upper left of the figure (after Meybeck and Helmer 1989)

Among potential inorganic tracers, conservatism is a function of reactivity (chemical stability) that, in turn, is a function of the chemistry of the tracer itself (e.g. transition metals, rare earth elements, radionuclides, stable isotopes), as well as how it is bound to the sediment. As such, the most conservative inorganic tracers are likely to be matrix-bound elements that include such constituents as Si, Al, Ti, Zr and the rare earth elements (e.g. Y, Yb, Gd, Sm). In terms of chemical stability, individual minerals (e.g. crystalline oxides such as rutile (TiO_2_) or sulphides such as pyrite (FeS_2_)) probably are on a par with matrix-bound elements because they usually are unaffected by the Eh/pH ranges found in most catchments. On the other hand, carbonate minerals (e.g. calcite/aragonite (CaCO_3_), dolomite (CaMg(CO_3_)_2_) that can be affected by changes within the normal pH range of some catchments would not be as stable. Typical trace elements, such as Pb, Zn, Cd, Cu and Ag, that are associated with sediment surfaces via sorption onto Fe oxide coatings, or organic carbon films, are likely to be more chemically reactive, and hence less conservative, than matrix bound constituents or individual minerals. The relative levels of chemical reactivity of a number of inorganic constituents that could serve as tracers (Fig. 4) often is related to their solubility (Meybeck and Helmer 1989). As such, the order of preference should range from lower to higher solubility (from the bottom right to the top left; Fig. 4). Whilst low solubility is a strong indicator of tracer conservatism, how the constituent is bound to a sediment particle also exercises a substantial amount of control. As an example, both Zn and Al have roughly the same solubility (Fig. 4); however, the former normally is sorbed to the surfaces of sediment grains whereas the latter normally is matrix bound. As a result, Zn concentrations are more likely to change as the sediment particle traverses a catchment as a result of sorption/desorption processes, whereas Al cannot. Hence, despite similar solubilities, Al is likely to be more conservative than Zn.

#### Biomarkers/organic tracers

**Fig. 5:**
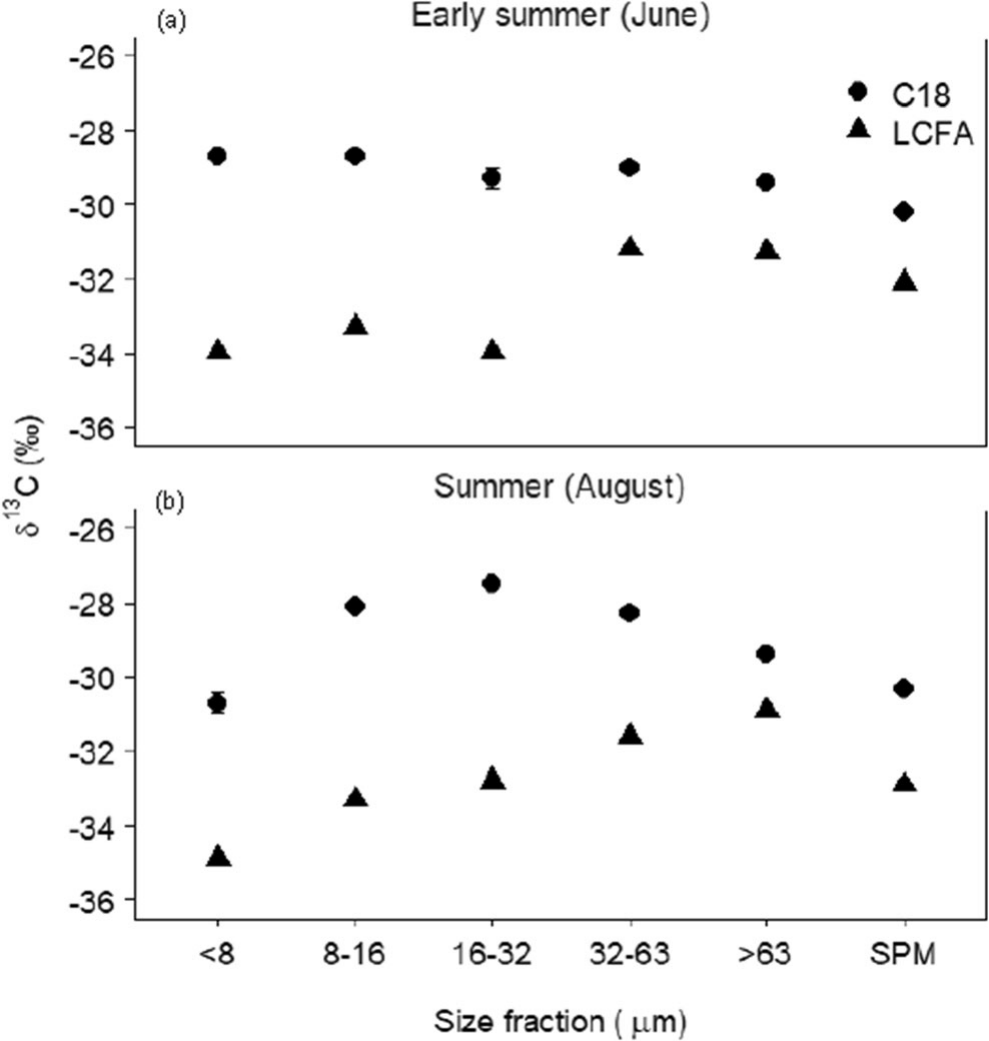
Carbon isotopic (δ^13^C) values of FAs (fatty acids) among size fractions in **a** early summer and **b** summer suspended particulate matter from the Yellow River, China. LCFA indicates the abundance-weighted average values of the δ^13^C_26 + 28 + 30_ FAs (after Yu et al. 2019)

Degradation of biomarkers (e.g. fatty acids, alkanes) over multiple scales during sediment generation and transport in a catchment can be responsible for non-conservative behaviour. Specifically, microbial mineralisation and re-synthesis transform the isotopic composition of biomarkers (Matsumoto et al. 2007). However, degradation-induced kinetic isotope fractionation can be independent of sources, carbon-chain length, grain size and sampling season. Given that sediment tracers are mainly associated with fine-grained minerals, hydrodynamic sorting processes can exert a significant influence on both the content and isotopic signature of biomarkers (Fig. 5) in river sediment (Laceby et al. 2017). Moreover, the biomarkers associated with larger grain-size fractions often associated with plant debris are typically younger compared with the finer fraction (Yu et al. 2019). Biomarkers in the finer fractions especially short-chain (< 20 C atom) are likely to be non-conservative due to their mineralisation and re-synthesis. Therefore, the conservative behaviour of the isotopic composition of biomarkers should be assessed carefully.

Plant-based biomarkers are promising for identifying land use-based sediment sources and their relative contributions to target sediment samples. These biomarkers are incorporated into the soil by higher plants from rhizodeposition and decomposition of organic matter and thus inherit the ^13^C signature of the vegetation from which they emanate (Reiffarth et al. 2016; Upadhayay et al. 2017). Because of their lower aqueous solubility, these biomarkers tend to be more resistant to microbial degradation than short-chain homologues in soil and sediment environments. Preservation is typically higher for alkanes relative to fatty acids (Cranwell 1981) and the latter are less likely to be adsorbed on soil particles due to lack of a functional group. It is important to acknowledge, however, that alkanes provide a means of understanding organic carbon origin rather than the source of soil mineral particles. Nevertheless, long-chain (> 20 C atom) *n*-alkanes and *n*-fatty acid stable isotope composition do provide insights into the origin of soil organic carbon from different land uses since these biomarkers are almost exclusively produced by higher plants (Dinel et al. 1990; Eglinton and Eglinton 2008; Upadhayay et al. 2020a).

**Fig. 6:**
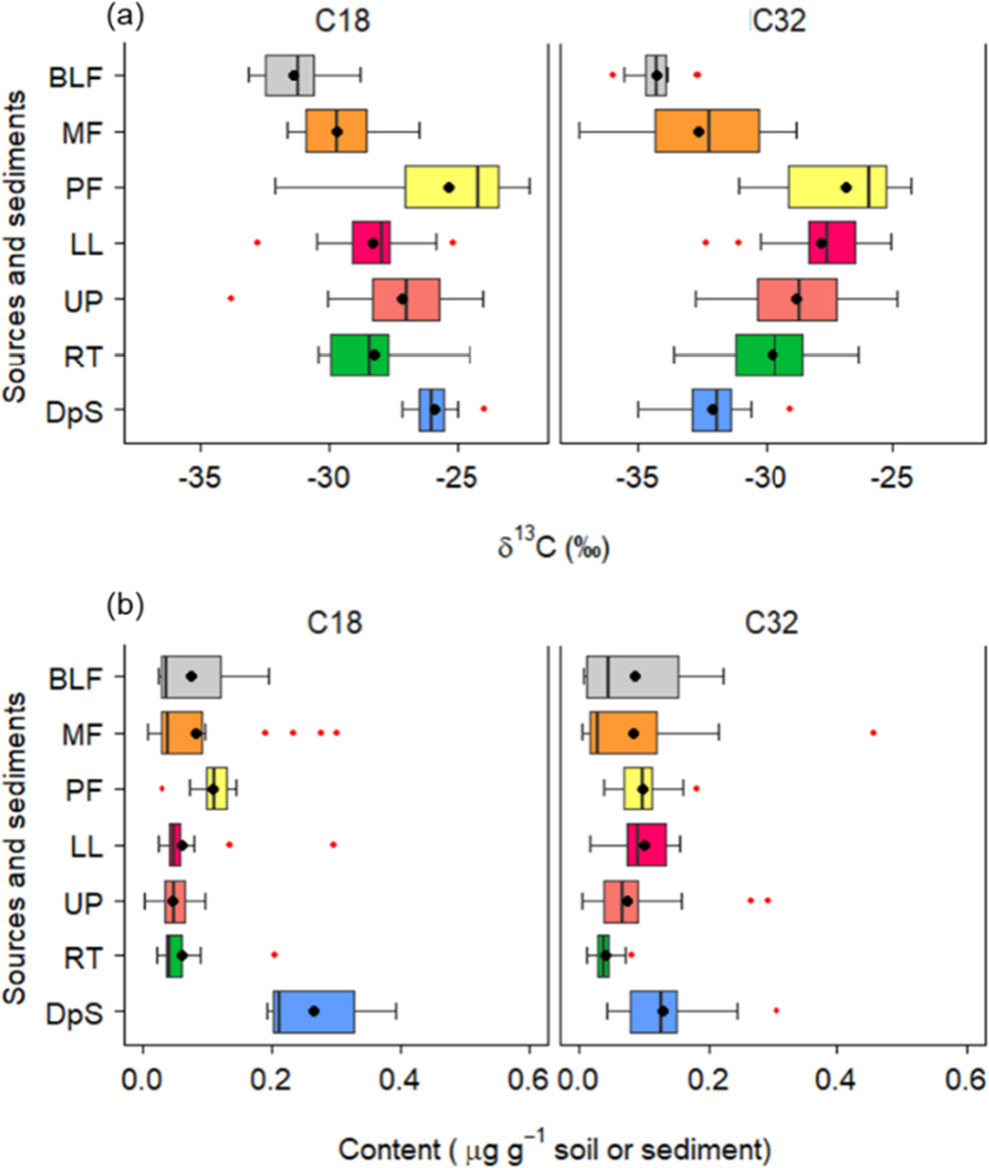
Distribution of δ^13^C values (**a**) and content (**b**) of representative short-chain (C18) and long-chain (C32) saturated FAs (fatty acids) in potential sediment sources (BLF, broadleaf forest; MF, mixed forest; PF, pine forest; LL, lowland agricultural terraces; UP, upland agriculture terraces; RT, road tracks) and target sediment (Dps) in the Chitlang stream, Nepal (after Upadhayay et al. 2018b)

It is still necessary to assess carefully the content of biomarkers to confirm conservative behaviour. This is important, since biomarker degradation and/or addition and associated isotopic transformation within catchment systems are highly complex, and the standard mathematical approaches used in sediment sourcing studies for tracer conservatism fail to detect such transformation. For illustrative purposes, Fig. 6 shows that the δ^13^C values of the C18:0 and C32:0 fatty acids in target sediment samples are within the corresponding ranges of the potential sediment sources, but the sediment C18:0 content is highly enriched compared with the sources (Fig. 6). This example clearly illustrates that the δ^13^C values of C18:00 in the land uses are obscured by other sources (e.g. algae) since short-chain fatty acids are also produced by microorganisms and aquatic plants. Additionally, the presence of bacteria can cause long-chain fatty acids to undergo degradation and to resynthesize as short-chain fatty acids (Fang et al. 2014). It should also be noted that fatty acid content can also be higher in target sediment samples compared with source materials due to fining of grain size in conjunction with hydrodynamic sorting processes and selective delivery. Nevertheless, investigators are advised to use long-chain saturated fatty acids for sediment source fingerprinting (Alewell et al. 2016; Upadhayay et al. 2017) and to take explicit account of sediment sorting (Yu et al. 2019).

#### Sedimentary deposits

The shortcomings associated with current conservatism tests are also relevant to fingerprinting studies using sedimentary deposits as target sediment. Many factors will affect the preservation of physical, inorganic and organic signatures of material deposited at the bottom of lakes and reservoirs potentially leading to alteration of the signatures and failure of the fingerprinting method. The bottom sediments themselves may be derived from at least four different sources: (1) from the atmosphere and (2) the catchment via rivers (both allogeneic sources), (3) from the lake itself through processes like bank erosion and organic matter production (authigenic) and/or (4) produced internally by organisms living within the upper few centimetres of the lake sediment (allogeneic). In the latter case, physical mixing of sediment and oxidation is likely to occur whereas sediment deposited below the biologically active layer is likely to be strongly reducing (e.g. Svensson et al. 2001; Baranov et al. 2016). In situ production of minerals (e.g. bacterial magnetite and greigite) and dissolution of minerals under strongly reducing conditions can confound the use of magnetic mineralogy for fingerprinting lake sediments (Foster et al. 1998, 2008; Sandgren and Snowball, 2001). Whilst many of the physical properties like particle size may not change through post depositional diagenesis, other chemical and/or organic properties may be significantly affected by dissolution and vertical diffusion. Dissolution and vertical diffusion appear to be Eh dependent, and the strength of reducing conditions determines which elements are likely to be more mobile (Fig. 4). In some cases, evidence for remobilisation has come from the use of ratios of potentially more mobile elements (e.g. Mn, Fe) to more stable elements (e.g. Al, Ti) (Reynolds et al. 2004).

### The physico-chemical basis for source discrimination

Despite significant developments in the variety of properties available along with almost exponential advances in un-mixing model capabilities, advances in our understanding of the physico-biogeochemical basis for source discrimination have arguably not kept pace. This, in part, reflects failure of the user community to engage with appropriate expertise. As more laboratories are equipped with relatively affordable instrumentation that can now generate a variety of fingerprint properties, it would be beneficial to focus some research attention on whether the hypothesized physico-biogeochemical foundation behind source discrimination is maintained during sediment generation, transportation and deposition processes and whether or not we need to consider moving beyond the current conservation tests and other statistical approaches and include more of the physico-biogeochemical science. Confirmation of the physico-chemical basis for source discrimination therefore remains an important knowledge gap for the selection of some fingerprint properties in composite signatures.

The historical development of the approach has been generally based upon the hypothesized discrimination provided by physical and biogeochemical parameters between one and more potential sediment sources. For example, the application of ^137^Cs and ^210^Pb_ex_ as effective fingerprinting properties was built upon the understanding that these fallout radionuclides have greater concentrations in surface soils relative to sub-surface soils, with cultivated soils exhibiting properties typically in between these two distinct end-members (Walling and Woodward 1992; Olley et al. 1993; Wallbrink et al. 1999). The utility of total organic carbon and total nitrogen concentrations is built upon a similar understanding in that these organic parameters typically decrease with soil depth (Walling et al. 1993; Mariotti and Peterschmitt 1994; Walling and Woodward 1995). Elemental geochemistry, colour and mineral magnetic parameters theoretically have tracers in their analysis suites that provide significant discrimination between potential soil types, dominant rock types or land uses (Walling et al. 1979; Hutchinson 1995; Collins et al. 1997; Poulenard et al. 2009). More recently, compound-specific stable isotopes have been selected based upon discrimination provided by the labelling of soils by degrading organic matter (Gibbs 2008; Reiffarth et al. 2016). For the majority of the properties utilized in the sediment source fingerprinting literature, there is, therefore, a known or hypothesized physical and/or biogeochemical basis for source discrimination that justifies their direct incorporation into un-mixing models (Fox and Papanicolaou 2007; Hatfield and Maher 2008; Olley et al. 2013; Cooper et al. 2015; Le Gall et al. 2017b).

Another approach to select fingerprinting properties is the utilisation of statistical procedures. In particular, the standard three-step approach to selecting fingerprinting parameters for use in end-member modelling includes some variant of a bracket test to remove non-conservative parameters followed by statistical tests (e.g. Kruskal Wallis H-test, DFA) to select an optimal group of fingerprints for modelling (Collins et al. 1996). The foundation of the statistical approach is based on incorporating a wide variety of fingerprinting parameters that should theoretically provide discrimination between the potential sources (Walling et al. 1993; Collins et al. 1996, 1998; Collins and Walling 2002). Collectively, the entire suite of fingerprinting parameters analysed is assumed to provide some physico-biogeochemical source discrimination and it is rightly argued that including more fingerprint properties in this three-step statistical procedure, should in theory, provide more accurate and consistent source apportionment results (Collins and Walling 2004; Walling 2005).

However, at times, it may be difficult to justify the physico-biogeochemical basis supporting the final selection of tracer properties obtained in each step of the widely used statistical procedure. This challenge is exemplified by the large number of potential tracing parameters often measured in routine analyses (e.g. ICP-MS, colour, mineral magnetics properties, radionuclides). For example, regarding the conservative behaviour of fingerprint properties, Smith and Blake (2014) excluded phosphorus (P) from the potential set of tracers on the basis of the risk of non-conservative behaviour during downstream transport. Kraushaar et al. (2015) combined additional physiochemical information on the sedimentation environment through water sample analyses, correlation analyses and a literature review to exclude multiple properties that may exhibit non-conservative behaviour from modelling (i.e. Na, Ca, K, Mg, Sr, ^40^K and TOC).

Koiter et al. (2013a, b) incorporated geological knowledge of their catchment rather than statistical tests to select properties. Laceby et al. (2015) and Batista et al. (2019) further demonstrated the utility of these knowledge-based approaches to trace, respectively, sediment derived from different geologies and soil types. Smith and Blake (2014) justified the selection of several geochemical properties to discriminate between surface and sub-surface sources on the basis of differences resulting from the weathering gradient varying with soil depth and soil surface contamination. Indeed, there has been a recent emphasis to justify the parameters selected by the three-step statistical procedure (e.g. Mukundan et al. 2010; Vale et al. 2016; Sellier et al. 2020) and investigators are encouraged to adopt this level of scrutiny, rather than relying on statistical solutions alone (Collins et al. 2017).

### Dissemination of sediment source fingerprinting results to key landscape actors

Despite the ongoing growth in publications utilising the sediment source fingerprinting approach, research into how landscape actors, implicated as a potential driver of excess sediment loadings, engage with the fingerprinting results is still lacking. As agricultural land is often a dominant source supplying excessive sediment loads to river systems around the world, fingerprinting data needs to be delivered directly to farmers and landowners alongside policymakers. However, farmers, catchment officers and policymakers may all have varied information requirements. Whilst many non-scientists may prefer simplistic summaries of scientific procedures and the findings, others may demand a detailed overview and qualification of the robustness associated with the employed research methodologies. Scientists may, however, struggle to communicate with farmers due to their different epistemologies, approaches, values, attitudes and experiential knowledge (Raedeke and Rikoon 1997; Tsouvalis et al. 2000; Eshuis and Stuiver 2005). Collaboration with trusted farm advisors may offer an effective delivery mechanism for disseminating results, as local one-to-one advice delivery is a highly effective mechanism for engaging with farmers and other landscape actors (Dwyer et al. 2007). When engaging with policymakers, the delivery of findings should occur during policy windows (Rose et al. 2017). Where source fingerprinting data contradict personal experiences or depict a lack of practical understanding, it is unlikely that they will be perceived as trusted or relevant by non-scientists (Eshuis and Stuiver 2005). Collaborative research with social scientists is warranted to determine whether a greater uptake of interventions for reducing excess sediment loadings may result from presenting the findings of source fingerprinting studies to local actors and policymakers.

## Emerging themes in sediment fingerprinting research and applications

### Novel tracers

Although suites of conventional properties (e.g. elemental geochemistry, radionuclides, mineral magnetics) have been used effectively to differentiate between major land use sources (e.g. cropland, forests, grassland) supplying sediment to receptors (Huon et al. 2013; Laceby et al. 2016; Lizaga et al. 2019), there is a fundamental need to increase the resolution of land use and land cover source discrimination. To achieve this goal, researchers have developed approaches to trace sediment sources based upon source soil and sediment compound-specific stable isotope (CSSIs) signatures (Gibbs 2008). To date, the CSSI approach has been limited to the use of fatty acids and alkane carbon isotopic composition to identify the contribution of fields under specific crop rotations (Blake et al. 2012; Mabit et al. 2018a, b), as well as sediment sources in forest plantations of Chile (Bravo-Linares et al. 2018) or mixed land use catchments (Upadhayay et al. 2018b). Integration of the δ^2^H values of long-chain alkanes and fatty acids in tracer sets has shown great potential for extracting additional information on sediment origin related to dominant vegetation types (Gao et al. 2011) and elevation gradients (Feakins et al. 2018). In addition, the isotopic composition and content of lignin-derived phenols and resin acids, as well as emergent biomarkers such as branched glycerol dialkyl glycerol tetraethers (brGDGTs) and methoxy-serratenes, have potential to distinguish the contributions of sediment from different land uses at catchment scale. Nonetheless, the development of this type of novel tracer technology faces several challenges including the inherent spatial and temporal variability of biochemical tracers (Reiffarth et al. 2016), the need to collect a large number of source samples and the potential importance of conducting multiple sampling campaigns to generate representative biomarker fingerprint properties of cultivated sources (Reiffarth et al. 2019).

To further increase the resolution of land cover tracing, another novel approach has been the potential use of *n*-alkanes, which are found in epicuticular waxes of leaves and are characterized by a slow degradation. These were first proposed for palaeoclimatic reconstructions (Eglinton and Eglinton 2008). More recently, this method was tested in a catchment in Southern Brazil to discriminate the sediment contributions supplied by *Pinus taeda* commercial plantations and those from native forests (Galoski et al. 2019). Biomarker analyses are expensive relative to many more conventional tracers and there remains a need to explore and confirm the incremental cost-benefits of their application in conjunction with traditional tracer types across a range of environmental settings with differing levels of source complexity.

With the rapid development of DNA sequencing technologies and the decrease in associated costs (Seymour 2019), environmental DNA offers another technique that may provide very detailed information on the vegetation types supplying sediment to river systems, with identifications that may go up to the species level (Evrard et al. 2019b). However, there remain several methodological challenges that require further research. In particular, upper organic and mineral topsoil layers were shown to be enriched in plant DNA compared with deeper soil horizons, which may complicate the use of this technique in catchments dominated by sub-soil erosion (Giguet-Covex et al. 2019).

Novel methods have also recently been proposed to refine the use of elemental geochemistry. For instance, the use of specific fractions of elements, generally obtained by sequential chemical fractionation using different extractors, has been promising for discriminating sediment sources, providing an excellent low-cost alternative to biomarkers or sediment DNA. For example, several P-fractions more sensitive to land use change have the potential to discriminate between sediment sources, especially in rural catchments where P is added via fertilizers (Tiecher et al. 2019). These authors demonstrated in a Brazilian rural catchment that whilst some operationally defined fractions of P in sediment sources are not conservative, some fractions (e.g. resin P, 0.5 M NaHCO_3_-P, 0.1 M NaOH-P and, total organic P) can be used in combination with geochemical tracers to improve source discrimination compared with using geochemical tracers alone. Whether this improved discrimination can be extended to more complex catchments with greater numbers of P sources remains to be tested. One approach that may help is to study the different forms of organic P in sediments, especially the inositol phosphates which are typically the most abundant forms of organic P in soils (Gerke 2015), via the use of NaOH-EDTA extraction and P-31 NMR (Turner et al. 2003; Cade-Menun and Liu 2014). Recent advances in identifying previously unconsidered high-molecular weight complex organic P compounds in NaOH-EDTA extracts (Mclaren et al. 2019) offer further potential to develop organic P approaches for improving sediment source tracing.

The use of the stable oxygen isotope ratio (^18^O) of phosphate (δ^18^O_p_) for tracing sources of phosphate has recently received increased attention. It works on the principle that the P–O bond in phosphate is stable under typical environmental temperature and pH and is broken only by enzyme mediated reactions, thus meaning that the isotopic source values remain the same unless biological activity results in exchange between oxygen in water and oxygen in phosphate. This enzyme mediated exchange between water ^18^O and phosphate ^18^O is generally assumed to primarily be driven by pyrophosphatase which leads to an ‘equilibrium’ δ^18^O_p_ value which can be predicted by water temperature and water ^18^O (Chang and Blake 2015) but which leads to the original source δ^18^O_p_ value being lost.

This methodology has been used successfully to trace the sources of wind-borne sediment (Gross et al. 2013, 2015, 2016). The application of this technique to trace sediment within the aquatic environment is, however, more complex (Pistocchi et al. 2017). The potential for multiple contributing sources, the overlap between different source δ^18^O_p_ ranges and the equilibrium range, within the water column, the risk of dissolved phosphate from non-sediment sources becoming adsorbed and the cycling of sediment P and exchange with water ^18^O, can all challenge the approach. Granger et al. (2017) found that within a moderately complex catchment, sediment source δ^18^O_p_ were similar to equilibrium values, meaning the source apportionment would have been impossible. Therefore, this method has limitations, but it may be effective in simple catchments with low sediment residence times and limited sources (Pistocchi et al. 2017).

Novel tracers have also recently been incorporated into sediment source fingerprinting through adopting approaches from other disciplines. For example, Nosrati et al. (2019) recently combined weathering indices (e.g. the chemical index of alteration, the weathering index of Parker, and the indicator of recycling) with geochemical tracers to investigate the spatial sources of suspended sediment in northern Tehran, Iran. Indeed, there is significant potential to develop novel tracers from other approaches utilized in Earth System sciences, including clay mineralogy, strontium (^87^Sr/^86^Sr) and neodymium (^144^Nd/^143^Nd) isotopes and rare earth element ratios (e.g. Nd/Yb, Gd/Yb, La/Sm) amongst others (Gingele and De Deckker 2005; Gholami et al. 2017a; Le Gall et al. 2017b). Some previous work has also illustrated the combined use of conventional sediment source fingerprints with artificial dual-signature (i.e. fluorescent and magnetic) tracer technologies to increase the spatial resolution of sediment (Collins et al. 2013) source information in both grassland (Collins et al. 2010) and arable (Collins et al. 2013) settings in the UK, and such approaches seem worthy of further testing, albeit in the context of the high costs of the dual-signature tracers which must be matched to the typical absolute grain size characteristics of the target sources and the high costs of the high strength magnets required for tracer sampling in the sediment receptor.

Although the development of novel tracers may increase our source discrimination capabilities, the importance of using multi-tracer (and multi-model) approaches was recently demonstrated as a means of averaging out the potential biases (e.g. source variability, particle size selectivity) associated with the different types of tracers (Uber et al. 2019). Accordingly, the integrated use of low-cost and high-resolution techniques should facilitate the analysis of a large number of samples to obtain reliable information on the variations in sediment source contributions (Evrard et al. 2019a). Spectroscopy, based on visible-near-infrared and shortwave-infrared, provides more than 70 physically based spectral features that have potential to trace sediment and sediment-associated organic matter sources (Collins et al. 2013; Brosinsky et al. 2014).

The potential of portable equipment including XRF devices to directly measure potential tracing properties in the field also opens novel avenues for research (Turner and Taylor 2018). Here, the work proposed the use of Rb measurements to correct the metal concentrations estimated in the field on fresh sediment in which the presence of interstitial water diluted the sediment mass and attenuated the incident X-rays. The full potential of other low-cost and high-resolution techniques including hyperspectral spectroscopy imaging remains to be explored (Butz et al. 2015; Aymerich et al. 2016; Jacq et al. 2019).

### Inclusion of concentration dependence for biomarkers in sediment un-mixing models

In the case of using isotopic abundance as a tracer (e.g. carbon, nitrogen), a significant source of uncertainty for source contributions is linked to not using biomarker content (i.e. biomarkers through which isotopic composition is derived) in un-mixing model formulation. Recently, land cover–dependent differences in the δ^13^C isotopic values of specific organic compounds (i.e. soil fatty acids, *n*-alkenes) have been used to estimate source contributions from different land use types without accounting for the effect of isotopic content on un-mixing model results (Gibbs 2008; Blake et al. 2012; Hancock and Revill 2013; Alewell et al. 2016; Brandt et al. 2016, 2018; Bravo-Linares et al. 2018; Mabit et al. 2018a, b). Here, the concentration-independent approach assumes an identical isotopic tracer content for all sources. This, however, has recently been shown to be the exception rather than the rule (Upadhayay et al. 2018a). In contrast, the alternative concentration-dependent approach accounts for the non-linearity of isotopic mixing in sediment by correcting for the content dependency within the un-mixing modelling. Different relative tracer contents in potential sources have a significant impact on the shape of the un-mixing modelling polygon (Hopkins and Ferguson 2012). Therefore, Upadhayay et al. (2018a) strongly recommended the use of concentration-dependency in isotope mixing regardless of the model framework, i.e. Bayesian or frequentist.

### The age and residence time of fine-grained sediment

Because sediment fingerprinting apportions the sources of delivered sediment, it cannot determine the residence time of the source sediment. Accordingly, we do not know if a portion of the target sediment travelled quickly from sources to the sampling point or whether it resided in storage zones for various time periods. As sediment travels from its origin to any sampling point, it can be deposited in storage on upland surfaces (i.e. colluvial slopes) (Smith et al. 2014) or in channel storage (i.e. the active channel bed, bars, and on floodplains) (Fryirs and Brierley 2001) for periods ranging from days or weeks to millennia (Lancaster and Casebeer 2007; Pizzuto et al. 2014; Hoffmann 2015). The time sediment remains in various storage units represent the residence time, whereas the sediment transit time is determined by the start and end points of where sediment enters and leaves the channel system.

It is difficult to know precisely the sites of sediment storage and transit times as sediment travels from source to sink. Therefore, investigations have examined the ‘age’ of sediment to infer its transit time from source to sink. To estimate sediment transit and residence times for sediment ‘age’, fallout radionuclides provide a marker for surface sediment (top soil) and provide useful chronometers, ^210^Pb_ex_, which can date sediment to ~ 85 years and ^7^Be to ~ 1 year (Mabit et al. 2008). Different methods have been developed to calculate sediment age, transit and residence times (Bonniwell et al. 1999). Matisoff et al. (2005) proposed a ^7^Be/^210^Pb_xs_ ‘chronometer’ to determine the age of sediment (*t*; Eq. 1) and the percentage of ‘new’ suspended sediment in the river (Eq. 2):

1 $$ t=\frac{-1}{\left({\lambda}_{7\ \mathrm{Be}-{\lambda}_{210\mathrm{Pb}}}\right)}1n\left(\frac{A}{B}\right)+\frac{1}{\left({\lambda}_{7\ \mathrm{Be}-{\lambda}_{210\mathrm{Pb}}}\right)}1n\left(\frac{A_0}{B_0}\right) $$

where *λ*_7Be_ and *λ*_210Pb_ are the decay constants of ^7^Be and ^210^Pb (d^−1^), *A* and *B* are the ^7^Be and ^210^Pb_ex_ activities in suspended sediment samples (Bq kg^−1^) and *A*_0_ and *B*_0_ are the ^7^Be and ^210^Pb_xs_ activities in rainfall (Bq L^−1^).

2 $$ \%\mathrm{of}`\mathrm{new}'\mathrm{sediment}=100\times \frac{\left(A/B\right)}{\left({A}_0/{B}_0\right)} $$

A decrease in the ^7^Be/^210^Pb_ex_ ratio can alternatively be explained by (i) an increase in the sediment residence time (given ^7^Be decay is faster than that of ^210^Pb_ex_) or by (ii) the dilution of sediment enriched in ^7^Be by sediment depleted in ^7^Be. Using the ratios of ^7^Be:^210^Pb_ex_, Matisoff et al. (2005) determined ages of suspended sediment ranging between 0 and ~ 300 days.

Dominik et al. (1987) using ^7^Be, ^210^Pb_ex_ and ^137^Cs suggested a 2-box age model for the alpine Rhône River, where topsoil particles travel slowly (800 to 1400 years) and a rapid box where high erosion moves particles between 1 and 220 days. Evrard et al. (2010) examined sediment residence time in a tropical watershed in central Mexico using ^7^Be, ^210^Pb_ex_ and ^137^Cs. Residence times were analysed with respect to a 2-box model similar to that of Dominik et al. (1987): a geologic box where soil is transported to the watershed outlet at time scales of 5000 to 23,000 years and a rapid box, where once in the channel, sediment travels over 50 to 200 days. Flood type, seasonality and land use all influence the timing and export of sediment (Evrard et al. 2010). The two-box model calculation (Dominik et al. 1987; Clarke 2008) requires the measurement of the fallout radionuclide input in a catchment and the corresponding radionuclide river output. The catchment surface is then subdivided into two boxes: (i) a ‘soil-box’ comprising the uppermost soil surface exposed to radionuclide fallout and (ii) a ‘river-box’ comprising the river and the nearby areas characterized by quicker transfers and shorter radionuclide residence times. The mean residence times of particles exposed to radionuclide fallout are calculated for each box. In the original work, Dominik et al. (1987) were confronted by the impossibility of totally solving the equations when using ^7^Be and ^210^Pb_xs_ and this challenge was resolved through the inclusion of ^137^Cs (Le Cloarec et al. 2007). Under certain environmental conditions (e.g. under acidic and ammonium-rich conditions or in saline environments), ^137^Cs is known to be chemically mobile and can be removed from soils in solution (Parsons and Foster 2011; Appleby et al. 2019).

However, the basic two-box approach has been challenged (Walling 2013a). To avoid potential differences in the ^7^Be/^210^Pb_ex_ ratios measured in rainfall and in recently eroded sediment, Evrard et al. (2016) recently proposed the assay of sediment collected in overland flow or using ‘edge-of-field’ samplers to characterize the source signature, instead of analysing rainfall. Another critique was that sub-surface material is theoretically sheltered from ^7^Be fallout (Walling 2013a). However, Hancock et al. (2014) demonstrated that, although this may be true for vertical sub-surface sources (e.g. gully sides and vertical channel banks), horizontal sub-surface sediment sources (e.g. gully floor material) may be exposed to rainfall and labelled with ^7^Be and ^210^Pb_ex_, whilst being depleted in ^137^Cs as shown earlier by Wallbrink and Murray (1993). To reflect this diversity in field settings, a distribution modelling approach using the concentrations of ^7^Be, ^137^Cs and ^210^Pb_ex_ measured in both source and sediment material was proposed to quantify the contributions of four source end-members (i.e. recently eroded surface, re-suspended surface, recently eroded sub-surface and re-suspended sub-surface) to sediment transiting the river (Evrard et al. 2016; Le Gall et al. 2018). However, one of the limitations of this approach is that it does not calculate an age or a residence/transit time.

**Fig. 7:**
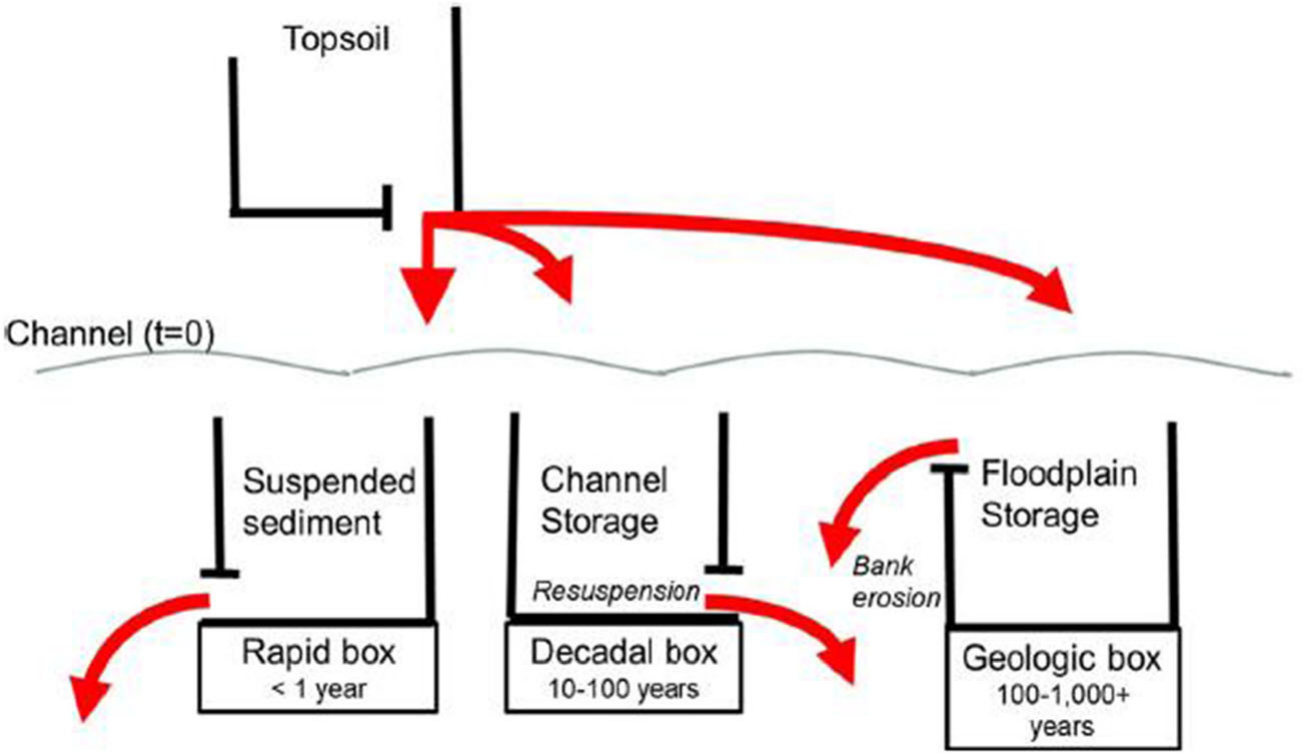
General model of sediment transit and residence times (after Gellis et al. 2019)

Gellis et al. (2016) apportioned sediment sources (channel versus land surface) using ^210^Pb_ex_ and the age (< 1 year) of fine-grained (< 63 μm) suspended and bed sediment using ^7^Be for 99 wadeable streams of the American Midwest. The findings suggested that channel sources dominate and that the age of the bed and suspended sediment ranged from 0 to 174 days. Both Matisoff et al. (2005) and Gellis et al. (2016) acknowledged that a shortcoming of their approach was that they could not determine whether the channel-derived sediment originated from streambanks or from older surface-derived sediment that had been in channel storage for several decades. In a follow-up study, Gellis et al. (2019) apportioned the sources of sediment in the agricultural Walnut Creek watershed, IA, USA, into channel banks and surface-derived sediment (pasture, prairie, cropland and unpaved roads) using sediment fingerprinting (elemental analysis) and fallout radionuclides (^210^Pb_ex_ and ^7^Be) to determine the age of the surface-derived portion of sediment (Fig. 7). A basic understanding of the hydrologic system is important in addressing possible problems with sediment residence times. For example, if a catchment is transport dominant, few sites of sediment deposition may occur, and sediment residence times may be low. In catchments that are transport limited, sites of deposition may be frequent and residence times higher. Geomorphic reconnaissance of the catchment (e.g. approaches such as Fitzpatrick et al. 2006) may be useful in qualifying the transport features of a catchment. Based on the different methods outlined above, fine-grained sediment transit and residence times are gradually being estimated around the world (Table 1).

**Table 1:** Sediment ages calculated with fallout radionuclides reported in the literature

Study area	Tracers	Sediment age(s)	Reference
Murrumbidgee R. (Australia)	^210^Pb_ex_	10 ± 5 years	Wallbrink et al. (1998)
Old Woman Creek (OH, USA)	^7^Be, ^210^Pb_ex_	46–79 days	Matisoff et al. (2005)
Weeks Bay (AL, USA)			
South Slough (OR, USA)			
Seine R. (France)	^7^Be, ^210^Pb_ex_, ^137^Cs	< 365 days; 4800–30,000 years	Le Cloarec et al. (2007)
Cointzio R. (Mexico)	^7^Be, ^210^Pb_ex_, ^137^Cs	50–200 days; 5000–23,300 years	Evrard et al. (2010)
Pleasant Valley (WI, USA)	^7^Be, ^210^Pb_ex_	123 ± 12 to 322 ± 144 days	Lamba et al. (2015)
Houay Pano (Laos)	^7^Be, ^210^Pb_ex_	8–158 days	Ribolzi et al. (2016)
Midwest (USA)	^7^Be	68% of streams < 100 days	Gellis et al. (2017)
Louroux R. (France)	^7^Be, ^210^Pb_ex_	0–215 days	Le Gall et al. (2017a)
Walnut Creek (IA, USA)	^7^Be, ^210^Pb_ex_	44–208 days;1–58 years	Gellis et al. (2019)
White Clay Creek (PA, USA)	^7^Be, ^210^Pb_ex_	1–110 days	Karwan et al. (2018)

Alongside dating fine-grained sediment in transit, current research continues to date fluvial sediment over the last two centuries using time specific tracers and the half-life of fallout radionuclides (Table 1). Time-specific tracers represent a given time or time period which can be natural or human caused. For example, ^137^Cs was delivered to the Earth’s surface following above ground thermonuclear bomb tests from the 1950s to 1970s with peak activity around 1963–1964. Generally, when delivered to the Earth’s surface, ^137^Cs binds strongly with fine-grained sediment and becomes a time marker of surface sediment, although some recent work identifies settings when this assumption is open to challenge (Appleby et al. 2019). Fluvial sediment that contains ^137^Cs activity indicates that a portion of the sediment was on the Earth’s surface during the period 1950s–1970s. Other time markers may dose sediment during a particular time period marked by an event such as an industrial waste disposal or discharge (Skalak and Pizzuto 2010), toxic spills (Lauer and Vengosh 2016) or radioactive spills (Graf 1996).

Techniques that can provide a chronology for timescales over the last 2–3 centuries are listed in Table 2. This is not an exhaustive list, and it is assumed that, because conventional ^14^C dating cannot be used for the last ca. 250 years due to fossil fuel combustion, only its fallout history after nuclear weapons testing could provide a practical chronology over more recent decades. However, alternatives (e.g. ^137^Cs, ^241^Am) are far more frequently used for dating sedimentary sequences, often in combination with ^210^Pb. Dating of recent sediments often benefits from changes in source properties over time; so, a factor that makes these changes a limitation for fingerprinting can become an advantage for dating. Spherical carbonaceous particles (SCPs) and heavy metals (HMs), for example, are both linked to industrialisation and atmospheric pollution. They have a fallout history in many industrialized countries that is well documented and can be used to identify known time periods, with SCPs first appearing in independently dated UK lake sediments around 1850 and the first notable rise in HMs around 1865. However, neither can be used for source tracing as concentrations of SCPs and HMs in potential sources (e.g. topsoil) will have changed throughout the history of fallout. Similar problems are encountered in using ^137^Cs, ^241^Am and ^14^C. All have a known temporal fallout history associated with atmospheric thermonuclear weapons testing and/or nuclear accidents. Several long-lived radionuclides such as ^238^U and ^235^U may have become enriched in agricultural topsoil where phosphate fertilizers are used as the North African rock phosphate from which a significant amount of fertilizer is derived often contains significant amounts of radioactive uranium (Sahu et al. 2014; Hassan et al. 2017). Neither of these isotopes or their daughters would be useful for fingerprinting over recent timescales in the same way that phosphorus could not be used directly for fingerprinting.

**Table 2:** Potential dating techniques for sediment over the time ranges from decades to centuries

Dating method	Half-life (years except where stated)	Recent timescale (years to decades)	Historic timescale (100s years)	Source	Reference
^7^Be	53.3 days	~ 1 year		Naturally produced radionuclide	Gellis et al. (2017, 2018); Matisoff et al. (2005)
^14^C	5730	From 1954			McGeehin et al. (2004); Hua (2009)
^241^Am	432.2	From 1954		Nuclear bomb fallout	Appleby et al. (1991); Arnaud et al. (2006)
^137^Cs	30.2	From 1954		Nuclear bomb fallout and nuclear accidents	Walling and Foster (2016); Foster et al. (2006)
^32^Si	~ 153		Last 500 years	Cosmogenic nuclide	Fifield and Morgenstern (2009); Finch et al. (2016)
^210^Pbex	22.3		Last 100–150 years	U-238 decay series	Appleby (2001); Walling and Foster (2016)
Luminescence			< 100–late Quaternary	Electron capture in quartz and feldspar sands	Thomas et al. (2007); Sawakuchi et al. (2016)
Varve chronologies			Holocene	Limited to environments with alternate seasonal freezing and thawing	
Heavy metals			Various	Atmospheric pollution, mining, smelting	Jones et al. (1991); Foster and Charlesworth (1996)
			since ca. 1800	urban transport related, toxic spillages	Maina et al. (2019); Lauer and Vengosh (2016)
SCPs			First rise ca. 1850	Industrial atmospheric pollution	Rose (2002); Rose and Appleby (2005)
Tephra			Late Holocene	Volcanic eruptions	Horowitz et al. (1995); Lowe 2011
Pollen			Last ca. 300 years	Local landscaping/vegetation disturbance records	Schottler and Engstrom (2006); Pittam et al. (2009)

Failure of ^210^Pb_ex_ and ^137^Cs to provide a reliable chronology for lake, reservoir and floodplain sediments over the last 100–150 years has also been reported. ^137^Cs is generally considered to be more chemically mobile in sediments than ^210^Pb (Appleby 2001; Fig. 4) and has been reported to be transported in the dissolved phase in significant quantities to lakes (Appleby et al. 2019). However, some lakes appear to have lower ^210^Pb_ex_ activities and inventories than predicted (Pittam et al. 2009), for which no plausible explanation has yet been found. Several factors, including acidic and ammonium-rich conditions, appear to favour ^137^Cs remobilisation as do highly saline environments in coastal environments (Foster et al. 2006). However, more mundane factors such as dredging can lead to the failure of a chronology unless the management history of individual sites is well documented. ^210^Pb dating has been reported to fail in lakes where surface runoff is negligible and thus sediment inputs are minimal, such as groundwater-dominated lakes in Florida as the parent isotope is present in groundwater and leads to ^210^Pb_ex_ activities lower than that predicted from the presence of its parent (Brenner et al. 2004). ^32^Si dating works in the same way as conventional ^14^C dating as it is a cosmogenic nuclide which falls out continuously and assimilates in the exoskeletons of organisms that use Si rather than Ca as the basic building block (diatoms). It is likely therefore to require the same calibration between ^32^Si age and calendar age as ^14^C. This is likely to prove much more problematic as other forms of silica are much rarer than the tree rings frequently used to calibrate ^14^C, but calibration may be possible against varved lake sediment sequences (Fifield and Morgenstern 2009). Tephra and exotic pollen rain can be regional and/or local in geographical extent, but there are searchable online databases for tephra such as Tephrabase (https://www.tephrabase.org/) that can find dates and locations of eruptions and their chemical signatures. Potential exotic pollen will normally require access to local estate records in order to establish planting history (e.g. Pittam et al. 2009) or make use of known periods of dramatic vegetation change (Schottler and Engstrom 2006). Varve chronologies are limited to cold climate environments where sedimentation couplets are produced alternately by spring snowmelt bringing minerogenic sediment into the lake basin and autumn dieback when organic matter is deposited. If it can be assumed that the varves are annual, the method can be used to help calibrate other dating methods (Fifield and Morgenstern 2009). The presence of coarse sedimentary layers relating to extreme precipitation events can also be used to help fine tune radiometric chronologies (Foster et al. 2007, 2019) whilst particle size analysis can help provide information on long-term palaeoflood records (Schillereff et al. 2014) as particle size distributions are rarely modified by post-depositional diagenesis. More recently, Foucher et al. (2020) have used high-resolution CT scanning to identify flood layers in sediment cores rather than stratigraphic or particle size data.

### Extending applications to other sediment-associated priority pollutants

Trace elements and organic pollutants have drawn attention as sediment-associated priority pollutants, and the identification of their source is therefore essential. The fine particle size fractions (typically < 63 μm) of sediment are known to be enriched in numerous contaminants (Owens et al. 2005), and an increasing number of studies are applying sediment fingerprinting techniques to quantify the source contributions or the dynamics of sediment-associated pollutants (Froger et al. 2018). For instance, Le Gall et al. (2018) analysed the fallout radionuclide contents in contaminated sediment mobilized during the exceptional flood on the Seine River in Paris (France) in 2016 and showed that this event mobilized, in particular, older contaminated sediment from an upstream tributary. Past contaminant concentrations are often reconstructed based on the analysis of sediment cores collected from lakes or in floodplains (Macklin et al. 1997; Desmet et al. 2012; Van Metre and Horowitz 2013). Importantly, the comparison of contamination levels in sediment samples collected at several locations in the river channel should systematically take into account the potential differences in particle size between samples and the local background levels when sediment-associated metals are investigated (Matys Grygar and Popelka 2016). Normalising the concentrations with geochemically insoluble elements may provide an alternative to sieving the samples to a specific particle size. A recent study conducted in a river of the Czech Republic suggested that Fe is preferable to Al and Ti for conducting such a normalisation (Tůmová et al. 2019). The analysis of metal contents in sediment is associated with several methodological debates associated with the use of total or partial digestion protocols (Dabrin et al. 2014). To avoid these problems and this time-consuming sample preparation, the increasing use of non-destructive X-ray fluorescence techniques may increase our capacity to provide high-resolution elemental profiles in sediment core profiles. However, these alternative techniques require demanding calibration protocols to provide absolute concentrations of sediment-associated contaminants (Lee et al. 2018).

Far fewer studies (Zou et al. 2015) have addressed the sources of organic pollutants such as polycyclic aromatic hydrocarbons (PAH)—a large group of persistent organic pollutants, which are among the most important priority pollutants based on a combination of their frequency, toxicity and potential for human exposure (ATSDR 2015) or polychlorinated biphenyls (PCBs) (Mourier et al. 2014). Combining multiple tools, including innovative methods (i.e. PAH correlations and sediment fingerprinting using fallout radionuclides), is essential to discriminate between legacy and contemporary PAH sources at the catchment scale (Froger et al. 2019). The main concerns regarding source identification using PAH are the similarities between multiple PAH sources (Li et al. 2003), the high photolytic degradation rates of PAH compounds, especially for those lighter compounds which may hamper their discrimination (Kim et al. 2009) and the uncertainties related to their potential non-conservative behaviour.

The ongoing development of novel techniques to conduct rapid, low-cost and non-destructive measurements with a high resolution on sediment core profiles will also significantly increase our capacities to quantify and understand the transfer of particle-bound contaminants. Recently, the use of hyperspectral sensors provided a way to analyse chlorophyll *a* to quantify changes in burnt organic matter levels in a lake draining a catchment exposed to forest fires (Van Exem et al. 2018). Collaborations between palaeoclimatologists, geomorphologists and hydrologists will stimulate novel methods and corresponding findings (Jacq et al. 2019).

### Incorporation of supportive spatial information to aid un-mixing model parameterisation

Whilst applications of sediment source fingerprinting procedures increase worldwide, there remains a need to explore the scope for enhancing conventional fingerprints and especially using additional tracers linking to the fundamental physical controls for sediment mobilisation and delivery and their potential spatial variation at landscape scale. In river drainage basins, chemical weathering plays an important role in geochemical cycles (Ohta and Arai 2007; Li and Yang 2010; Shao et al. 2012; Guo et al. 2018). As a result, the eroded materials transferred by rivers preserve valuable information on land surface weathering, erosional status and processes and climatic variation. In particular, it is possible to explore the intensity of chemical weathering based on the information recorded in river sediment (Ohta and Arai 2007; Goldberg and Humayun 2010; Carter et al. 2015; Guo et al. 2018). Here, weathering indices can be used to indicate the intensity of chemical weathering processes using the degree of elemental mobility caused by the depletion of mobile relative to immobile elements during recycling (Price and Velbel 2003; Nadlonek and Bojakowska 2018). Because weathering indices reflect complex interactions between the climate regime, lithology, soil development, tectonism, topography, vegetation cover and anthropogenic activity including land use (Gibbs 1970; Meybeck 1987; Oliva et al. 2003; Li and Yang 2010; Shao et al. 2012; Négrel et al. 2015), their consideration as potential sediment source tracers provides a means for physically grounded source discrimination (Motha et al. 2003; Mohammadi Raigani et al. 2019; Nosrati et al. 2019). To date, however, limited research effort has been invested in the scope for using weathering indices alongside more conventional fingerprint properties. Instead, much research has continued to focus on testing different combinations of conventional tracers in composite fingerprints and the corresponding potential contrasts in predicted source apportionment (Owens et al. 2016; Collins et al. 2017; Smith et al. 2018; Tang et al. 2019). Nevertheless, weathering indices might provide useful tracers for source discrimination and apportionment since they reflect the propensity for soil erosion, meaning that a sub-basin with more exposed highly erodible formations might be expected to have more sediment mobilisation (Garzanti et al. 2016).

There are numerous weathering indices (Table 3) such as enrichment factors and indices of geochemical maturity (e.g. the resistant index, the oxidative index) based on soil and sediment geochemistry, and these can be used to interpret weathering history. CIA (chemical index of alteration) is potentially useful as a tracer since it provides a basis for identifying chemical changes caused by weathering of alumino-silicate minerals (Motha et al. 2003; Haddadchi et al. 2013; Owens et al. 2016). In general, higher CIA values suggest more weathering of silicates (Shao et al. 2012). A mean CIA value for the suspended sediment in select global rivers has been estimated at 72.1 (Li and Yang 2010), compared with a corresponding value of 79.7 for the upper continental crust (Taylor and McLennan 1985).

**Table 3:** Information on chemical weathering indices

Chemical weathering index	Formulation*	References
Chemical index of alteration (CIA)	Al_2_O_3_/(Al_2_O_3_ + CaO + Na_2_O + K_2_O) × 100	Nesbitt and Young (1982); Ohta and Arai (2007); Li and Yang (2010); Buggle et al. (2011); Shao et al. (2012); Guo et al. (2018).
Modified weathering potential index (MWPI)	((K2O + Na_2_O + CaO + MgO)/(SiO_2_ + Al_2_O_3_ + Fe_2_O_3_ + K_2_O + Na_2_O + CaO + MgO)) × 100	Vogel (1975)
Weathering index of Parker (WIP)	(2Na_2_O/0.35 + MgO/0.9 + 2K_2_O/0.25 + CaO/0.7) × 100	Parker (1970); Price and Velbel (2003); Guo et al. (2017, 2018).
Product index (PI)	100*SiO_2_/SiO_2_ + TiO_2_ + Fe_2_O_3_ + Al2O_3_)] × 100	Ruxton (1968)
Chemical index of weathering (CIW)	Al_2_O_3_/(Al_2_O_3_ + CaO + Na_2_O) × 100	Harnois (1988)
Plagioclase index of alteration (PIA)	(Al_2_O_3_ − K_2_O)/(Al_2_O_3_ + CaO + Na_2_O − K_2_O) × 100	Fedo et al. (1995); Price and Velbel (2003); Buggle et al. (2011)
Recycling ratio (RI)	CIA/WIP	Garzanti et al. (2016); Guo et al. (2017, 2018)
Silica-alumina ratio index (SA) or Ruxton ratio (RR)	SiO_2_/Al2O_3_	Ruxton (1968)
Vogt ratio (VR)	(Al_2_O_3_ + K_2_O)/(MgO + CaO + Na_2_O)	Guan et al. (2001)
Si–Ti Index	(SiO_2_/Al_2_O_3_)/((SiO_2_/TiO_2_) + (SiO_2_/Al_2_O_3_) + (Al_2_O_3_ + TiO_2_))	Jayawardena and Izawa (1994)
Silica-sesquioxide ratio (Kr)	SiO_2_/(Al_2_O_3_ + Fe_2_O_3_)	Moignien (1966)
Alumina-sodium to calcium oxide ratio (ACN)	Al_2_O_3_/(Al_2_O_3_ + K_2_O + Na_2_O)	Harnois and Moore (1988)
Alumina to potassium-sodium oxide ratio (AKN)	Al_2_O_3_/(K_2_O + Na_2_O)	Harnois and Moore (1988)
Alkaline ratio (ALK)	(K_2_O/(K_2_O + Na_2_O)) × 100	Harnois and Moore (1988)
Hydration coefficient (Hc)	H_2_O/(K_2_O + Na_2_O + CaO + MgO)	Ng et al. (2001)
Leaching coefficient (Lc)	SiO_2_/(K_2_O + Na_2_O + CaO + MgO)	Ng et al. (2001)
Residual coefficient (Rc)	(Al2O_3_ + Fe_2_O_3_)/(K_2_O + Na_2_O+ CaO + MgO)	Ng et al. (2001)
Sesquioxide content (SOC)	Al_2_O_3_ + Fe_2_O_3_	Irfan (1996)
R_2_O_3_ ratio	Al_2_O_3_ + Fe_2_O_3_ + MgO + K_2_O + Na_2_O + CaO + TiO_2_ + P_2_O_5_ + ZnO + MnO + Rb_2_O)/MgO	Duzgoren-Aydin et al. (2002)
Index of desilication (ID)	SiO_2_/R_2_O_3_	Singh et al. (1998)
Loss of ignition (LOI)	LOI content in weight of sample heated in a range 900–1000 °C	Sueoka et al. (1985)

*All the weathering indices are calculated based on molecular weights of elemental oxides (Garzanti 2016), corrected for Ca by considering the ratio of CaO to Na_2_O (McLennan 1993)

Previous literature demonstrates that some early work by Motha et al. (2003) determining the sources of suspended sediment in a forested catchment, in south-eastern Australia, concluded that the CIA was useful for separating gravel-surfaced roads from three other sources categorized as undisturbed forest, harvested areas and ungravelled roads. More recently, Nosrati et al. (2019) showed that spatial variation in some weathering indices was consistent with corresponding spatial variation in suspended sediment concentrations measured for different catchment spatial sources during runoff events in a mountainous setting in Iran. On this basis, geochemical tracers were combined with the weathering indices to fingerprint sub-basin spatial suspended sediment sources. Similarly, Mohammadi Raigani et al. (2019) used a spatial sediment source fingerprinting approach to provide sediment provenance information in a mountainous agricultural catchment in western Iran. This study suggested that weathering indices potentially offer useful information for helping inform the pre-selection of fingerprint properties.

**Fig. 8:**
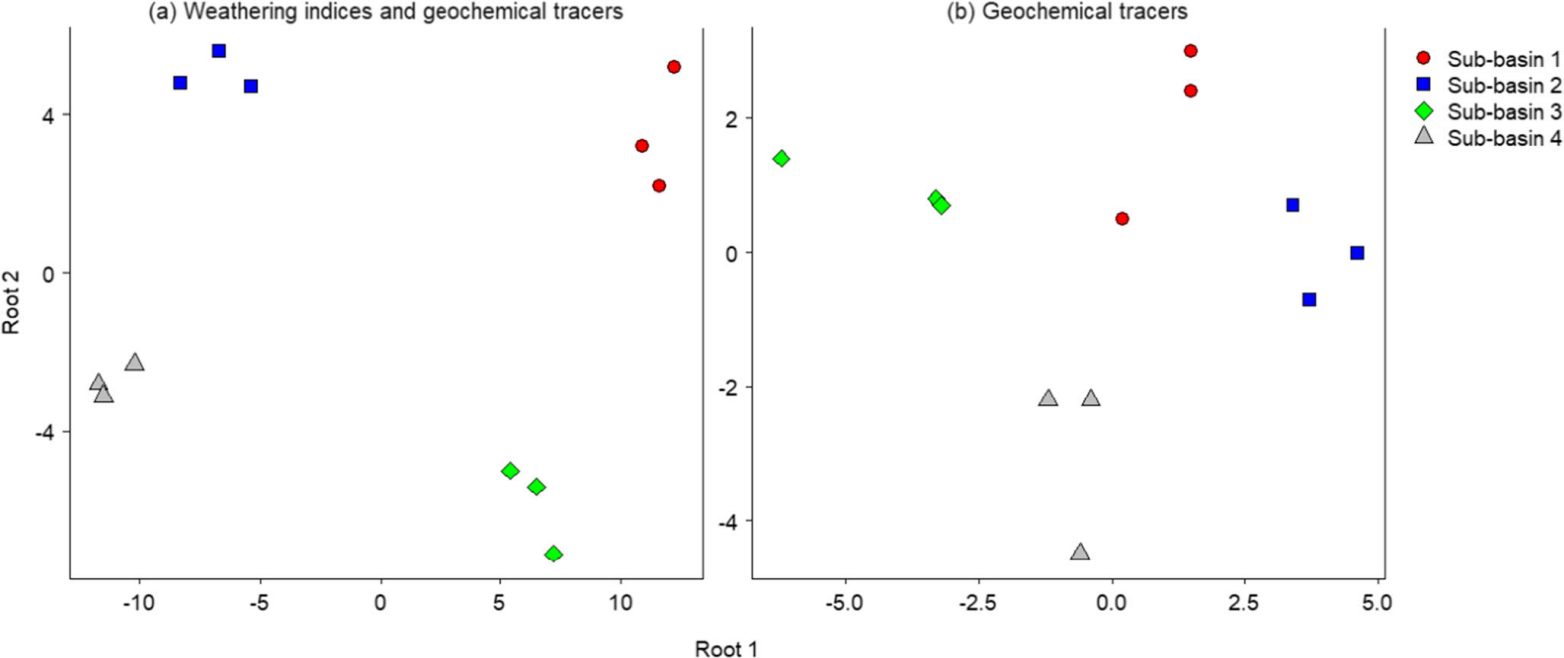
Visual comparison of the discrimination of tributary sub-catchment spatial sediment sources using two different composite signatures: left hand plot—weathering indices and geochemical tracers (CIA, WIP, IR, Cu, Fe, Mn, Sr, Zn) and right hand plot—geochemical tracers only (Cu, Fe, Mn, Sr, Zn) (after Nosrati et al. 2019)

Composite signatures should comprise different types of tracers to maximize discriminatory efficiency. In the context of using weathering indices to provide supportive spatial information for source discrimination and apportionment, it is advisable that any geochemical tracers included are not also used in the estimation of any weathering index selected for a final signature, since the requirement for using independent tracers in composite fingerprints should not be overlooked. As an example, Fig. 8 compares the discriminatory efficiency of two composite signatures for discriminating sub-basin spatial suspended sediment sources from the recent study of Nosrati et al. (2019). The two signatures comprised the combined use of weathering indices and geochemical tracers (CIA, WIP, IR, Cu, Fe, Mn, Sr, Z) and geochemical tracers only (Cu, Fe, Mn, Sr, Zn). In this case, the results clearly demonstrated the increasing discriminatory power by adding the weathering indices. Future research should expand assessment of the application of weathering indices in combination with more conventional fingerprint properties for additional hydroclimatic settings. With regards fingerprint property conservation, longitudinal sediment sampling in large drainage basins would also permit weathering indices to be used to explore any potential evolution of tracer transformation risks across scales and, again, this should be explored in future work.

However, it is important to acknowledge that care is needed in interpreting geochemical indices. Differences in rock chemical composition, especially those that have been affected by localized changes such as igneous intrusions or other metamorphic processes, can lead to differential deterioration rates not only across different lithologies but also within similar lithologies (Sayyed 2014). Furthermore, exposure to environmental stresses, such as temperature changes and precipitation, can lead to both localized strengthening of the rock surface (e.g. the formation of crusts and case hardening; McAlister et al. (2003); Viles and Goudie (2004)) and deterioration through the preferential draw-through of internal moisture (Mol 2014) and exposure to increased temperatures (Smith et al. 2011b). These localized alterations in the mineral composition, as well as the differential deterioration of the rock, surfaces as a function of mineral composition and exposure to environmental stresses (Weiss et al. 2004). Therefore, a combined approach of geological mapping (Laceby et al. 2015; Pulley et al. 2017a, 2017b) geomorphological assessments of rock deterioration (Mol and Viles 2012; Pola et al. 2014) and landscape connectivity (Navratil et al. 2012) is required to investigate potential sediment provenance, further diagenesis and material weathering post-removal from the parent rock. Significant geomorphological events (e.g. landslides; Pickup and Marks 2000) also need to be mapped. This does, however, open up new avenues of investigation, where sedimentologists work closely with geologists and geomorphologists.

### Application in aeolian sediment provenance studies

Aeolian sediments, including wind storm dust, sand and loess, are emitted from land surfaces in a range of different climatic settings. These sediments can smother crops, reduce visibility thereby impacting on traffic safety and result in long-term health problems due to a decline in air quality, especially in urban areas (Goossens 2003). A range of techniques has been used to investigate key dust sources for targeting remedial action including, amongst others, data mining with ensemble or individual models (Gholami et al. 2020a, b), remote sensing (Filonchyk et al. 2018), comparison of strontium (^87^Sr/^86^Sr) or neodymium (^143^Nd/^144^Nd) ratios (Yang et al. 2008; Saitoh et al. 2011), rare earth elements (Dukes et al. 2018) and detrital zircon ages or heavy mineral composition (Garzanti et al. 2013; Fyhn et al. 2019). Since 2016, however, there has been a growing interest in applying sediment source tracing procedures using linear mixing models (Liu et al. 2016; Gholami et al. 2017a; Wang et al. 2017; Zhao et al. 2020; Niu et al. 2020), Monte Carlo simulation (Gholami et al. 2019b), Bayesian approaches (Gholami et al. 2017b, 2019a) and generalized likelihood uncertainty estimation (GLUE) model (Behrooz et al. 2019; Telfer et al. 2020; Gholami et al. 2020c). A key step that differs from applications tracing fluvial sediment sources concerns the need to take explicit account of the dominant wind direction to inform source material and target sediment sampling. Another key step in aeolian sediment source fingerprinting is enclosing the study area since a fluvial catchment boundary cannot be used. Here, the study area boundary is based on the wind direction and dominant size fraction in the target sediment sample (e.g. dust, loess or wind-blown sand). The dominant size fraction in dune sand samples is typically 100–300 μm, and this size fraction typically originates from proximal sources. In the case of finer aeolian sediment (i.e. dust), potential sources include both proximal and regional (distal) sources. Here, investigators can use remote sensing and a HYbrid Single-Particle Lagrangian Integrated Trajectory (HYSPLIT) model to identify distal potential sources for sampling (Gholami et al. 2020c).

### Informing landscape management through focused integration of sediment fingerprinting

In the vast majority of cases, sediment fingerprinting is applied as a ‘stand-alone’ tool, but it is important to acknowledge the benefits of integration with other approaches as a means of advancing either the scientific understanding of landscape scale sediment systems or to provide resource managers with the specific information for targeting interventions for sediment management. Sediment budgets are amongst the most common landscape management frameworks for describing the inputs, transport, storage and export of sediment in catchments either qualitatively or quantitatively and integration of sediment fingerprinting data can provide valuable input terms (Walling and Collins 2000; Walling et al. 2001, 2006).

Fingerprinting approaches have been similarly integrated with deterministic models (e.g. SWAT) to describe watershed-level hydrological processes that affect sediment erosion and transport within catchments to inform management strategies and prioritize sub-watershed implementation of best management practices (BMPs) for erosion control by respectively identifying sources of suspended sediment and evaluating the amount of surface runoff generated (Palazón et al. 2014, 2016; Malhotra et al. 2018). These examples highlight that sediment fingerprinting approaches may inform the need to consider additional processes in both qualitative and quantitative models/frameworks as a result of insights gained; they also may assist with deterministic model evaluation, calibration or parameterisation (e.g. Collins and Anthony 2008; Stromqvist et al. 2008). Since many deterministic models are sector or source-specific (e.g. agriculture only), the integration of process-based modelling and source fingerprinting can permit examination of the spatial mismatch often hampering the efficacy of targeting BMPs to a particular source but with an overarching aim of improving water quality at a downstream monitoring station. Here, the source apportionment data can be used to correct the modelled efficacy of the source-specific BMPs (Collins et al. 2014). Recently published spatial strategies for sediment source fingerprinting (Pulley et al. 2017a, b; Blake et al. 2018; Haddadchi et al. 2019) can assist in the integration of such work and process-based modelling since the latter discretizes landscapes into sub-units. Given the controls on the sediment cascade imparted by hillslope-to-channel connectivity, the more spatially refined source tracing procedures better support integration with process models since all source tracing results are scale-dependent (Koiter et al. 2013a, b).

### Application of sediment fingerprinting to wildfire impacted landscapes

Sediment fingerprinting approaches are emerging in the characterisation of a broad range of potential impacts and associated risks resulting from climate change-exacerbated landscape disturbances such as wildfires, which can significantly impact ecological and societal goods and services from regional water resources. Wildfires generally reduce infiltration and increase surface runoff (DeBano et al. 1998), thereby promoting erosion and downstream propagation of sediment and associated contaminants. Elevated suspended sediment concentrations are common after wildfire (relative to those observed in unburned watersheds), especially at high flow conditions (Debano et al. 1998; Silins et al. 2009; Smith et al. 2011a); associated nutrients such as phosphorus are thus also frequently elevated (Lane et al. 2008; Emelko et al. 2016). The availability of limiting nutrients such as phosphorus can promote downstream productivity that leads to biofilm development and bed stabilisation in rivers; it ultimately also can result in substantially more erosion of bed sediments in wildfire-impacted watersheds (Stone et al. 2011). Increased nutrient availability can also initiate cascading ecohydrological effects on the abundance and diversity of aquatic organisms at several trophic levels (Silins et al. 2014; Martens et al. 2019) and threaten the uninterrupted provision of safe drinking water (Emelko et al. 2011; Writer et al. 2014).

Investigations integrating sediment fingerprinting and complementary process studies have highlighted both initial and more sustained impacts of wildfire on erosion and sediment delivery processes. To date, such efforts have largely focussed on assessing changes in post-fire sediment sources (e.g. surface versus bank erosion); most of these studies have reported a moderate to strong initial shift from gully and bank sediment sources to surface sources, followed by gradually declining erosion from surface sources, as vegetation begins to recover after wildfire (Blake et al. 2006, 2009; Wilkinson et al. 2009; Smith et al. 2011b, 2012, 2013a). These effects can vary across hydroclimatic regions where post-fire fluvial processes and potential impacts on post-fire bank stability and sources may be important when sufficient precipitation and snowmelt are available to drive erosion and sediment transport (Owens et al. 2006, 2012). Whilst fewer studies have assessed the longevity of fire effects on fine sediments, Stone et al. (2014) used composite geochemical fingerprinting to show that downstream transport of fine sediments from a large fire were still evident 6 to 7 years post-fire. In this case, more than 80% of the downstream sediment geochemical contribution in a large river basin was generated from 14% of the upstream landscape affected by a wildfire that occurred 6 to 7 years earlier.

The application of sediment fingerprinting to explore fire effects on both sediment sources and downstream fate of post-fire sediments is promising, but several challenges remain regarding source identification. For example, whilst a range of physical and geochemical properties of sediment have been previously used as tracers in wildfire studies, the results have often been inconclusive because of the differential effects of fire on sediment properties. There remains a critical need to identify and rigorously quantify sediment properties unique to the effects of wildfire on landscapes with different vegetation and source materials to apply these tracers to improve the discriminatory power of currently available fingerprinting procedures.

### Development of open access fingerprinting software to engender robust data processing by the growing user community

Due to the increasing complexity of sediment source fingerprinting procedures associated with data analysis for confirming source discrimination and un-mixing modelling for source ascription, a few software packages have been produced to facilitate wider uptake of the fingerprinting approach. SIFT (SedIment Fingeprinting Tool; Pulley and Collins 2018) contains the fundamental parts of the decision-tree published by Collins et al. (2017), with key features including the reclassification of a priori source groups using cluster analysis and maps of differences in tracer concentrations and sediment provenance results to provide a visual examination of sediment provenance. Similar to SIFT, The Sediment Source Assessment Tool (Sed_SAT; Gorman-Sanisaca et al. 2017) also contains all of the fundamental parts of a fingerprinting methodology and includes corrections for sediment organic matter content and particle size, tests for outliers and data transformations. Fingerpro (Gaspar et al. 2019) includes a Kruskal-Wallis rank sum test, range test, the option to merge source groups, box plots and bi-plots of source and of sediment tracer concentrations, a linear discriminant analysis and an un-mixing model. Whilst parts of the methodology such as the use of virtual mixtures or multiple composite fingerprints are not included by default, a user can run the model multiple times or input mixture values into the software to achieve these needs.

Despite the dominance of the frequentist approach for un-mixing modelling, Bayesian statistical inference is increasingly popular among the international sediment source fingerprinting community (Upadhayay et al. 2017; Davies et al. 2018). Here, for instance, MixSIAR is an inclusive, rich and flexible state-of-the-art Bayesian tracer un-mixing model capable of including fixed and random effects as covariates that explain variability in mixture proportions (Stock et al. 2018). Recently, Upadhayay et al. (2018b) and Blake et al. (2018) used a deconvolutional approach in MixSIAR to deal with tracer variability in sources and the complexities of catchment systems. It should be noted that the Bayesian approach provides a robust basis for combining prior information with data to produce the posterior distributions of source contributions (Upadhayay et al. 2020a, b). These authors used a non-subjective empirical data-derived informative prior, i.e. a sediment connectivity index, with compound-specific stable isotopic tracers to improve sediment source apportionment at catchment scale. Importantly, however, these models are not always fit-for-purpose; therefore, it is the user responsibility to decide on prior data treatment and the un-mixing modelling framework.

## Conclusion

Global uptake of sediment source fingerprinting continues to accelerate, but there is now a stark concomitant need to standardize key procedural details to ensure greater harmonisation, comparability and high standards. Both the desire to investigate sediment sources in more study catchments and the need to improve our understanding on a number of outstanding challenges, again with a vision of assisting procedural harmonisation, mean that applications are likely to continue to expand. Here, it remains important for the international community to devise and enact a collaborative process to drive harmonisation and standardisation of procedural details. This should include sharing datasets, model code and research facilities and replicating critical experimental work across scales to address the outstanding issues reviewed herein. This paper provides an up-to-date overview of the remaining scientific challenges and emerging trends for sediment fingerprinting and will hopefully assist the convergence of methodological detail to ensure that the approach is still seen as a valuable means of understanding and managing fine-grained sediment problems observed across the world. Our specific recommendations for future critical research topics are:

- To examine for all tracers and across environments and scales therein, the spatial and temporal variability of tracers and the corresponding implications for robust sampling strategies
- To examine for all tracers and across environments and scales therein, conservatism with a view to refining knowledge-based pre-selection
- To examine for all tracers, the physico-chemical basis for source discrimination
- To explore in more depth, stakeholder preferences for the presentation of source fingerprinting results.
